# Supplementary material for: Autonomous bioisosteric replacement for multi-property optimization in drug design
Source: Nat Commun. 2026 Jul 27;17:9113. doi: 10.1038/s41467-026-75512-9 (PMC13518832; doi:10.1038/s41467-026-75512-9)
Supplement: Supplementary file 1 [file 41467_2026_75512_MOESM1_ESM.pdf]

Supplementary Information for:

Autonomous Bioisosteric Replacement for Multi-Property Optimization in Drug Design

Hyeongwoo Kim,[a]‡, Seokhyun Moon[a]‡, Wonho Zhung,[a] Shinwoo Kim,[a] Jaechang Lim[b] and Woo Youn Kim*[abcde]

[a]Department of Chemistry, KAIST, 291, Daehak-ro, Yuseong-gu, Daejeon, 34141, Republic of Korea.

[b]HITS Inc., 124, Teheran-ro, Gangnam-gu, 06234, Seoul, Republic of Korea.

[c]Kim Jaechul Graduate School of AI, KAIST, 108, Taebong-ro, Seocho-gu, Seoul, 06764, Republic of Korea.

[d]Department of AX, KAIST, 291, Daehak-ro, Yuseong-gu, Daejeon, 34141, Republic of Korea.

[e]InnoCORE AI-CRED Institue, KAIST, 291, Daehak-ro, Yuseong-gu, Daejeon, 34141, Republic of Korea.

*Corresponding author; E-mail: wooyoun@kaist.ac.kr.

‡These authors contributed equally to this work.

Contents

1 Suppl. Note 1. Architecture details 2
- 1.1 Node and edge features . . . 2
- 1.2 Embedding of complete molecules and molecular fragments . . . 2

2 Suppl. Note 2. Training details 3
- 2.1 Post-processing the constructed MMP dataset for training . . . 3
- 2.2 Training with negative sampling strategy . . . 3
- 2.3 Hyper-parameters for training DeepBioisostere . . . 4

3 Suppl. Note 3. Generation details 5
- 3.1 Pre-processing for generation . . . 5
- 3.2 Optimization process with DeepBioisostere . . . 5

4 Suppl. Note 4. Dataset statistics 6
- 4.1 Property distributions of the training dataset . . . 6
- 4.2 Statistics of the fragment library . . . 6

5 Suppl. Note 5. Additional results and discussions 9
- 5.1 Ablation study on architecture design . . . 9
- 5.2 Analysis with additional test sets . . . 10
- 5.3 More statistical analysis on multi-property control scenarios . . . 12

6 Suppl. Note 6. Generated molecule examples and analysis 13
- 6.1 Property distributions of multi-property controlled molecules . . . 13
- 6.2 More examples of bioisosteres for an uncommon fragment . . . 14

7 Suppl. Note 7. Details on the *in silico* hit-to-lead workflow 15
- 7.1 Used structure-based 3D molecular generative models . . . 15
- 7.2 Target selection criteria and statistics of generated molecules . . . 15
- 7.3 Additional results . . . 16

1 Suppl. Note 1. Architecture details

1.1 Node and edge features

A molecule and molecular fragments are represented as nodes and edges corresponding to atoms and bonds, respectively. Supplementary Table 1 summarizes the atom and edge features used in this work, $\hat{\mathbf{a}}_i \in \mathbb{R}^{F_a}$ and $\hat{\mathbf{e}}_i \in \mathbb{R}^{F_e}$. A feature with more than two available options is represented in a one-hot vector corresponding to its category, while a feature with only two options (bool type) is encoded into a bit vector. Hydrogen atoms are represented implicitly by the feature of 'Num hydrogens'. Ring structures, including aromatic rings, are crucial information in drug discovery, so we included both features and represented them as one-hot vectors.

**Node feature**	**Available options**
Period	1, 2, 3, 4, 5, 6, else
Group	1, 2, 13, 14, 15, 16, 17, 18, else
Degree	0, 1, 2, 3, 4, 5, 6, else
Formal chage	-2, -1, 0, 1, 2, else
Num hydrogens	0, 1, 2, 3, 4, else
Chirality[a]	cw, ccw, unspecified, else
Hybridization	s, sp, sp^2, sp^3, sp^3d, sp^3d^2, else
Aromaticity (bit)	true, false
Ring member (bit)	true, false
BRICS type[b]	1, 2, 3, 4, 5, 6, 7, 8, 9, 10, 11, 12, 13, 14, 15, 16, 17
**Edge feature**	**Available options**
Bond order	single, double, triple, aromatic
Stereochemistry	Z, E, cis, trans, any, none
Conjugated (bit)	true, false
BRICS-allowed[c] (bit)	true, false

[a] cw: clockwise (R configuration). ccw: Counter-clockwise (S configuration).
[b] The BRICS type is assigned only for dummy atoms to represent the BRICS types of broken bonds. Refer to supplementary section 1.2.
[c] Representing whether a particular bond can be broken based on the BRICS rules.

Supplementary Table 1: Atom and edge features and their available items used in this work. All information is encoded into either one-hot or bit vectors to be concatenated into node and edge feature vectors.

1.2 Embedding of complete molecules and molecular fragments

One of the key characteristics of DeepBioisostere is a hierarchical architecture that handles a molecule at the levels of both atom and fragment. For embedding a single molecule, it is first analyzed by the BRICS rules to yield irreducible fragments. Afterward, a whole graph AMPN runs on the original molecular structure, while a subgraph AMPN operates on each internal fragment. The hidden vectors from each AMPN network are then aggregated into the fragment level and concatenated to fragment embedding vectors; $\mathbf{f}_n^0 = [\mathbf{w}_n \| \mathbf{s}_n]$. We note that input for subgraph AMPN comprises molecular fragments with dummy atoms to designate allowed attachment sites. We defined the atom feature vector of the dummy atoms to carry information only about the BRICS types and no information about other features in Supplementary Table 1. To match the dimension, a zero vector is concatenated into the feature vectors of non-dummy atoms. The resulting feature dimensions of a node and edge are $F_a = 66$ and $F_e = 12$, respectively. An example of this process is illustrated in Supplementary Fig. 1.

As a complete molecule is embedded by the embedding networks of DeepBiosostere, molecular fragments from the fragment library are embedded to be used as inputs for the insertion fragment selection module, θ_I. The nodes of a molecular fragment are embedded by whole graph and subgraph AMPN networks and

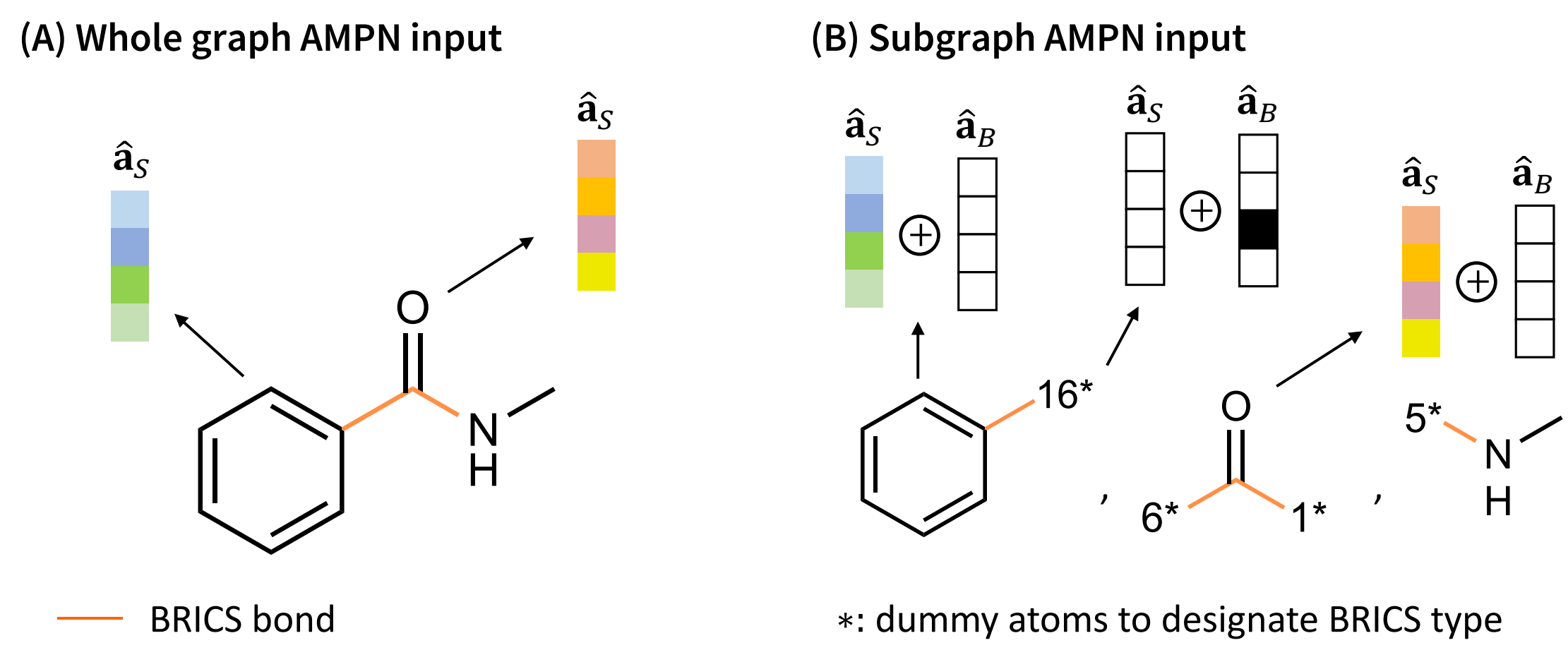

Supplementary Figure 1: $\hat{\mathbf{a}}_S$ is a concatenation of one-hot vectors to represent structural atom features, and $\hat{\mathbf{a}}_B$ is a one-hot vector to designate the BRICS type of a dummy atom. The numbers in front of the dummy atom symbol, $*$, indicate the corresponding BRICS types of broken attachment sites.

aggregated into a single embedding vector for that single fragment, the same way where the fragment-level embeddings of a complete molecule are obtained. The AMPN networks for a molecular fragment share their parameters with the AMPN networks for embedding a complete molecule. In this manner, molecular fragments are embedded without any bias on whether they come from a molecule or a fragment library. This implementation enables the AMPN networks to encounter diverse molecular fragments so that they can behave stably when a rare fragment takes part.

2 Suppl. Note 2. Training details

2.1 Post-processing the constructed MMP dataset for training

DeepBioisostere consists of three main modules, which are trained simultaneously with the training dataset we constructed. Each data point comprises an MMP for which two chemical modifications can be identified in opposite directions. For the respective modification, the differences in the molecular properties are used to construct the property control condition, **C**. Hence, two modifications with opposite directions and property control conditions are defined from each MMP data point. Removal and insertion fragments in the retrieved modifications are gathered to organize a fragment library to be used in the training and sampling processes.

2.2 Training with negative sampling strategy

In this work, we formulated three loss functions for the three main modules in the form of binary cross-entropy loss: $\mathcal{L}_{remove}$, $\mathcal{L}_{insert}$, and $\mathcal{L}_{attach}$. With a typical strategy for multi-class classification problems, a model is trained to predict an extremely sparse vector for each training data point—only a single 1 among about 200,000 zeros. Adopting the embedding vectors of the entire fragment library is a possible option to resolve this sparsity problem, but it might cause a substantial inefficiency in the training phase to evaluate tens of thousands of fragments for every data point. Thus, we implemented a negative sampling inspired by Seo et al., where only tens of negative answers are randomly sampled from a pre-defined fragment library and used to calculate the training loss for every single data point and epoch.[1] We implemented different negative sampling methods for each main module since their expected functions are subtly different; it is enough for the θ_R and θ_A modules to prioritize an answer over a few other options originating from the original molecule, but the θ_I module should find out a proper fragment from a large number of external options from a pre-defined fragment library.

In the case of the θ_I module, for insertion fragment selection, we employ a random sampling to choose

a few negative samples (the quantity as a hyperparameter was set to 20 in this work) from the fragment library except for a ground-truth fragment for each data point, and the selected fragments are treated as them as negative answers. We note that only fragments possessing the same number of attachment points as the ground truth and conforming to the BRICS rules for attachment with the remaining fragments are exclusively considered. In the cases of the θ_R and θ_A modules, responsible for removal fragment and attachment orientation selection, we designate all non-answer options from the original molecule as negative samples. Particularly, the internal fragments with no more than 12 atoms in the original molecule and different combinations of attachments are regarded as negative samples. We sampled negative answers with these intricate criteria since, in the sampling phase, we would allow the DeepBioisostere model to choose an option from the available choices that adhered to the specified criteria for each module.

We noticed that the frequency of removal and insertion fragments is highly imbalanced; that is, the times each fragment appears as a ground truth are different. Let's suppose fragments A and B appear 10,000 and 1 times as positive samples, respectively, and 1 time as negative samples for insertion fragment selection. In this training scenario, a model is vulnerable to being trained to prefer fragment A regardless of the situation it encounters. Thus, overlooking this imbalance problem might lead to a trained model that depends solely on the frequency data for crucial decisions. For this purpose, we adopted two training strategies — reflecting the frequency information on the negative sampling for the insertion fragment and employing a weighted sampler to resolve the imbalance problem in the removal fragment. The negative samples for insertion fragment selection are sampled with a probability rate weakly proportional to the fragment frequency. Formally,

$$p(f^I_{\text{neg}} = f_i) \propto \text{freq}^{\alpha_1}_{I,i}, \tag{1}$$

where f_i is a fragment in the pre-defined fragment library, $\text{freq}_{I,i}$ is the occurrence of f_i in the fragment library, and α_1 is a hyper-parameter to control the dependency of negative sampling on the frequency data. The hyper-parameter α_1 can take a value in the range of [0,1]. Here, 0 designates a sampling from the uniform distribution, while 1 corresponds to a sampling with probabilities linearly proportional to the frequencies.

The imbalance problem in removal fragments is handled by a weighted sampling strategy. Different from the insertion fragment selection, we utilize substructures of the original molecule other than the ground truth as negative samples. This implies that we cannot adopt an external fragment as a negative sample for a certain data point. Hence, resolving the imbalance problem in removal fragments should work at the level of training data sampling. We employ WeightedRandomSampler implemented in PyTorch that generates mini-batches by sampling a number of MMP data points from the training dataset with given weighted probabilities.[2] We introduce an additional hyper-parameter α_2 to control the dependency of the training data sampling on the removal fragment frequencies. The probability of each data point to be sampled is as follows:

$$p(\text{data} = \text{data}_i) \propto \text{freq}^{\alpha_2}_{R,i}, \tag{2}$$

where $data_i$ is a data point in the training dataset, $\text{freq}_{R,i}$ is the occurrence of the removal fragment of $data_i$ in the training dataset, and α_2 is a hyper-parameter to control the dependency of the weighted sampler on the frequencies. The hyper-parameter α_2 can take a value in the range of [-1,0]. In this context, -1 compels the model to encounter all removal fragments an equal number of times, while 0 allows all training data to be sampled with an equal probability, leading to an unbalanced sampling in terms of removal fragments.

2.3 Hyper-parameters for training DeepBioisostere

The hyper-parameter setting for the reported DeepBioisostere model is summarized in Supplementary Table 2. The model hyper-parameters are used mainly for the embedding layers of AMPN and FMPN and the three main selection modules. Many training hyper-parameters are ones for the ReduceLROnPlateau learning rate scheduler implemented in the PyTorch package that gradually reduces the learning rate for convergence stability.[2] The training hyper-parameters α_1 and α_2 are responsible for controlling the dependency of the DeepBioisostere model on fragment frequencies in the fragment library and MMP dataset, respectively. Since the occurrence of removal and insertion fragments in the datasets are highly imbalanced, they are very crucial to finding novel bioisosteres.

Model param.	Descriptions	Values
F_a^h	node hidden dimension	256
F_e^h	edge hidden dimension	256
$N_{\text{AMPN,whole}}$	num layers of whole-graph AMPN network	4
$N_{\text{AMPN,sub}}$	num layers of subgraph AMPN network	4
N_{FMPN}	num layers of FMPN network	2
N_{θ_R}	num layers of removal fragment selection module, θ_R	3
N_{θ_I}	num layers of insertion fragment selection module, θ_I	3
N_{θ_A}	num layers of attachment orientation selection module, θ_A	3
**Training param.**	**Descriptions**	**Values**
B_d	batch size for data points	512
B_f	batch size for fragment library embedding	512
lr	initial learning rate	2×10^{-4}
lr_{min}	minimum learning rate[a]	1×10^{-7}
$\text{lr}_{\text{factor}}$	lr decaying factor[a]	0.5
$\text{lr}_{\text{patience}}$	lr decay tolerance epochs[a]	10
$\text{lr}_{\text{threshold}}$	threshold for measuring the new optimum based on loss decrease[a]	1×10^{-3}
p_{dropout}	dropout rate	0.2
α_1	exponent to the negative sampling rate of insertion fragment[b]	0.5
α_2	exponent to the weighted random sampler for removal fragment[b]	-0.5
N_{neg}	number of negative samples to train θ_I module	20

[a] Parameters for the ReduceLROnPlateau scheduler implemented in PyTorch.[2]
[b] Please refer to Eq. (1) and Eq. (2) for more detailed definitions.

Supplementary Table 2: Descriptions of the hyper-parameters and their values used in this work.

3 Suppl. Note 3. Generation details

3.1 Pre-processing for generation

DeepBioisostere performs property optimization with two inputs: a molecule to be modified, M, and a property control condition, $\boldsymbol{C}$. First, the molecule is pre-processed based on the BRICS rules before being queried to the DeepBioisostere model. All BRICS bonds inside the molecule are identified so that its fragment-level graph representation is obtained. Then, substructures of the fragment-level graph are enumerated as potential removal fragments. Here, only a substructure that meets two strict criteria on its size is identified as an allowed removal fragment: 1) the number of heavy atoms should be less than or equal to 12, and 2) the number of heavy atoms should be less than or equal to the total number of atoms of remaining fragments. All the allowed fragments are to be examined by the removal fragment selection module θ_R in the later step.

3.2 Optimization process with DeepBioisostere

After pre-processing, the DeepBioisostere model encodes the given molecule along with the property control condition $\boldsymbol{C}$ into fragment-level embedding vectors. An embedding vector of each allowed fragment, $\mathbf{h}_f$, is obtained by summing the embedding vectors of internal irreducible fragments, following the equation (8) in the main text. Then, the removal fragment selection module θ_R utilizes the embedding vectors $\{\mathbf{h}_f\}$ to examine the likelihood of each allowed fragment as the equation (9) in the main text. From the multinomial probability distribution of the allowed fragments, the DeepBioisostere model performs random sampling until a pre-defined number of samples are obtained. We note that this process allows duplication so that the numbers of sampled removal fragments follow the multinomial distribution estimated by the model.

The DeepBioisostere model then explores the fragment library to find suitable insertion fragments for each removal fragment to achieve the property control condition. For each removal fragment, insertion

fragments that satisfy the following two criteria are evaluated by the model: 1) the number of attachment sites, or fragmented sites, should be the same as that of the removal fragment, and 2) the BRICS types of the sites are allowed to be attached to the remaining fragments. All the passed fragments are evaluated for their likelihood by the insertion fragment selection module θ_I as the equation (10), and highly ranked insertion fragments are greedily selected. It means that for each removal fragment, top-k insertion fragments are chosen according to the number of samples determined in the previous step. This greedy selection is possible since DeepBioisostere is a generative model that estimates the explicit likelihood of a generated sample.

Lastly, DeepBioisostere determines the attachment orientations of the selected insertion fragments. This step is performed as explained in the main text; only BRICS-allowed combinations are prioritized by the DeepBioisostere model. For each removal and insertion fragment pair, the top-1 attachment orientation is selected as an assembly template to which the DeepBioisostere model refers to build a final molecular structure.

4 Suppl. Note 4. Dataset statistics

4.1 Property distributions of the training dataset

For a more reliable estimation of the DeepBioisostere model's performance on multi-property control tasks (Section 2.2 in the main text), we present distributions of molecular properties and property deviations for MMPs. From the training dataset, we utilized all resulting molecules to illustrate the former distribution, while for the latter, all MMPs were employed. Fig. 2 shows the distributions for four properties considered in our property control tasks: the logarithm of the octanol-water partition coefficient ($\log P$), molecular weight (MW), quantitative estimate of drug-likeness (QED), and synthetic accessibility score (SAscore).

Notably, the QED difference distribution in Fig. 2(C) shows that increasing QED by 0.1 and 0.2 corresponds to 1.03 and 2.05 standard deviations, respectively - indicating that achieving a 0.2 QED gain is more challenging than increasing it by 0.1. For SAscore (Fig. 2(D)), our property control objectives include a 0.5 decrease (1.67 STD) and an even more demanding 1.0 decrease (3.32 STD). Yet, fewer than 0.05% of pairs in the training dataset exhibit SAscore differences above 1.0. Due to this statistical nature, QED or SAscore-control tasks become significantly more challenging.

4.2 Statistics of the fragment library

We conducted a statistical analysis of the number of bioisosteres corresponding to a fragment to illustrate the potential challenges that database mining approaches might face in the discovery of novel bioisosteres. Since our dataset only consists of MMPs, we first counted the number of bioisosteres for each fragment in our fragment library and then constructed a distribution based on these counts. Fig. 3(A) illustrates the resulting distribution, which includes 145,959 fragments from our library. As noted in the main text, a significant portion of the fragments, over 100,000, has ten or fewer bioisosteres, while only 1,269 fragments have 400 or more bioisosteres. Furthermore, Fig. 3(B) highlights 10 fragments with the highest number of bioisosteres. It comes with no surprise that benzene derivatives or methoxy groups, which are among the most common moieties, are found in these fragments, in contrast to the triazolo pyridazine moiety mentioned in the main text.

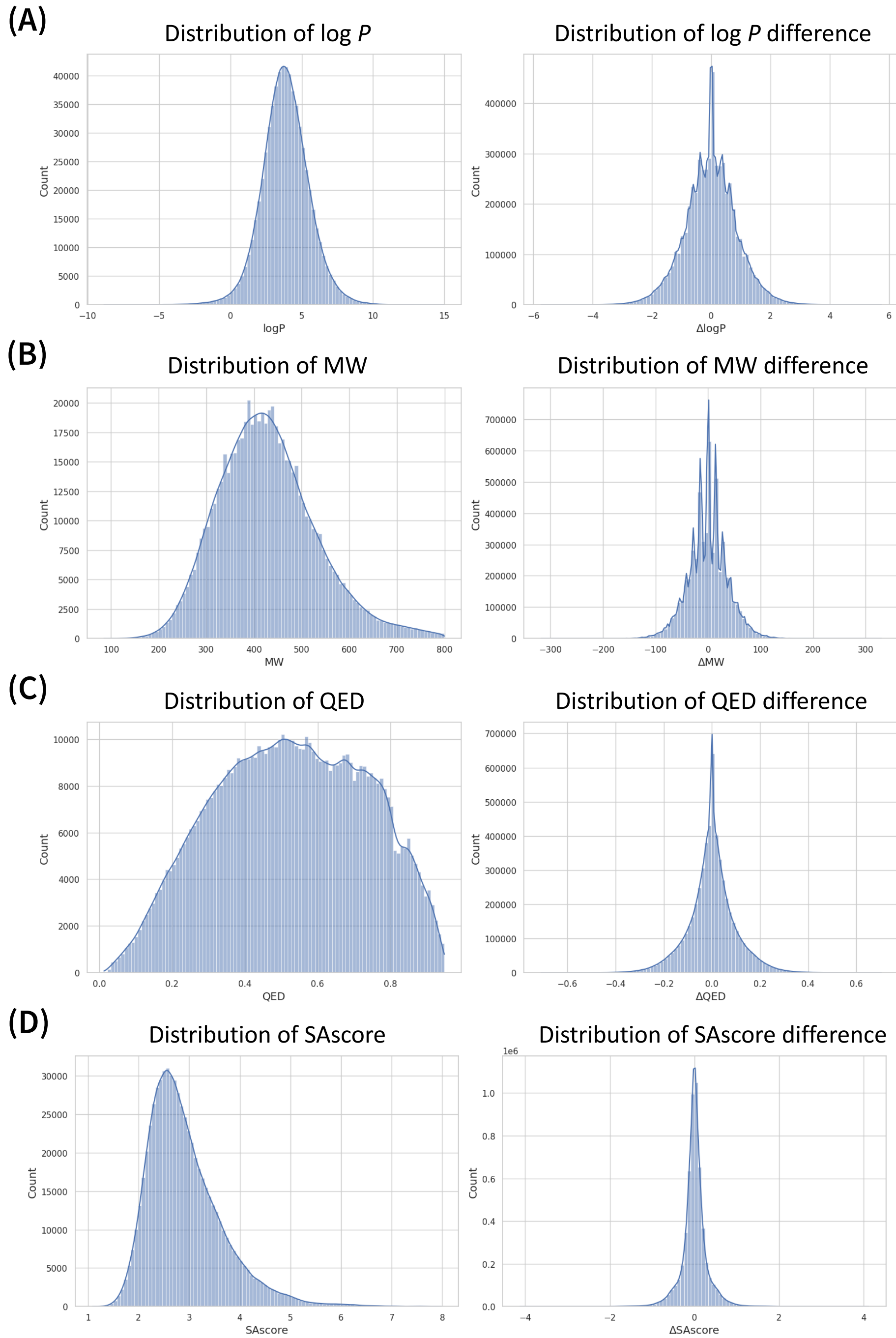

Supplementary Figure 2: Distributions of (A) $\log P$, (B) MW, (C) QED, and (D) SAscore in our MMP database. For all properties, the first and second columns represent the molecule property and MMP pairs' property deviation distributions, respectively.

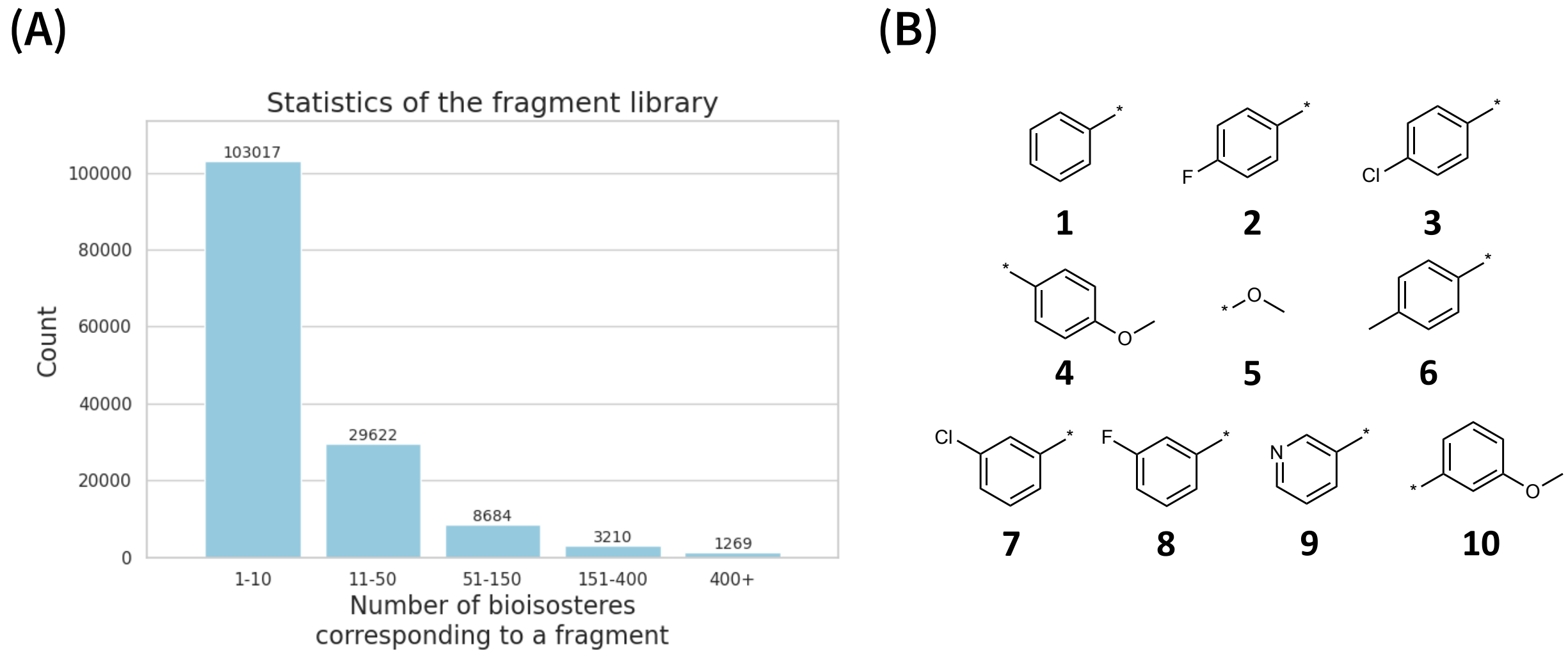

Supplementary Figure 3: Statistical analysis of the fragment library we constructed. (A) Statistics of the numbers of bioisosteres for each fragment in our fragment library. (B) The top 10 molecules that have the top 10 largest numbers of bioisosteres based on our MMP database.

5 Suppl. Note 5. Additional results and discussions

5.1 Ablation study on architecture design

We performed an ablation study on the architectural design of DeepBioisostere for the multi-property control task described in Section 2.2. We trained a DeepBioisostere model ablated with the subgraph AMPN during the original molecule embedding procedure; in this model the subgraph AMPN is not shared across the molecule and fragment library embeddings, but only used for the fragment library. In the original DeepBioisostere model, outputs from the whole graph AMPN and subgraph AMPN are concatenated and used as an initial hidden vector for the FMPN. However, since the subgraph AMPN is not used for original molecule embedding in the ablated model, we added a linear layer between AMPN and FMPN to upscale the whole graph AMPN outputs to fit the FMPN dimension, ensuring the ablated model has an equivalent number of parameters.

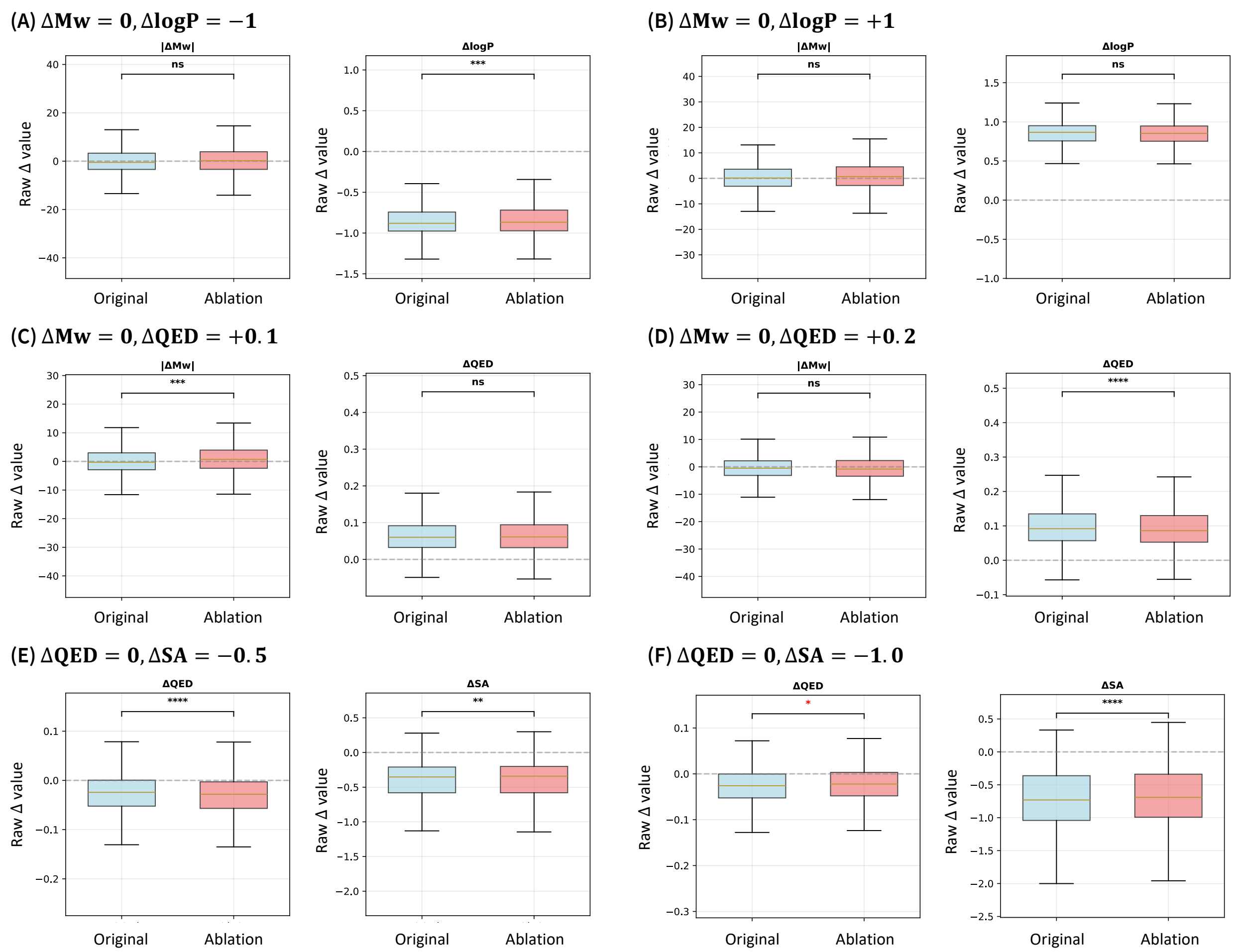

Supplementary Figure 4: Paired t-test results to compare multi-property control performances; ns $P > 0.1$, $*P < 0.1$, $**P < 0.05$, $***P < 0.01$, and $****P < 0.001$. Black stars designate the original model statistically achieves higher performance, and red ones mean the ablated model exhibits better results. Used input property control conditions are shown in sub-titles. (A,B) Scenario where MW and $\log P$ are modulated simultaneously. (C,D) Scenario where MW and QED are modulated simultaneously. (E,F) Scenario where QED and SA are modulated simultaneously.

As in Section 2.2, we applied the ablated model to the same test molecules with the same property control conditions, generating distinct sets of modified molecular structures. We then applied paired t-tests

that examines the property improvements or maintenance for each original molecule. We applied one-sided paired t-tests for each evaluated property within a multi-property control task (see Supplementary Table 3 for details).

The results in Supplementary Fig. 4 and Supplementary Table 3 show that the DeepBioisostere model generally outperforms or matches the ablated model across the six multi-property control tasks. While statistically significant differences ($P < 0.05$) are observed in 6 out of the 12 individual hypothesis tests, these results should be interpreted in the context of paired multi-property optimization, where each task simultaneously evaluates two coupled molecular properties with inherent trade-offs. In most tasks, the original model achieves either statistically significant improvement in one property while maintaining comparable performance in the other, indicating that the original architecture preserves more favorable trade-offs between jointly controlled molecular properties.

Although the boxplot distributions in Supplementary Figure 4 appear visually similar, the paired differences reveal small but consistent shifts across molecules (e.g., Δ log$P$: +0.0134 ± 0.1503 in Task A), which lead to statistically significant results under the paired t-test. These results highlight that the significance arises from consistency across paired samples rather than large absolute differences.

A mixed outcome is observed for the Δ QED = 0 and Δ SA = -1.0 condition (Fig. 4(F)), where the ablated model exhibited better QED maintenance with weak significance ($P = 8.23 * 10^{-2}$), whereas the DeepBioisostere model improved SA with much stronger significance ($P = 4.42 * 10^{-9}$).

Task	n	Property	Objective	Original	Ablation	Paired difference	P value	Preferred
A	995	MW	minimize $\|\Delta\text{MW}\|$	4.5212	4.6471	-0.1259 ± 3.5714	0.133	ns
		log$P$	maximize $-\Delta\log P$	0.8419	0.8285	0.0134 ± 0.1503	0.00245	Original
B	995	MW	minimize $\|\Delta\text{MW}\|$	4.6666	4.7833	-0.1166 ± 3.9336	0.175	ns
		log$P$	maximize $\Delta\log P$	0.8330	0.8325	0.0005 ± 0.1332	0.453	ns
C	979	MW	minimize $\|\Delta\text{MW}\|$	3.7701	4.0906	-0.3205 ± 3.5469	0.00240	Original
		QED	maximize ΔQED	0.0682	0.0689	-0.0006 ± 0.0302	0.254	ns
D	979	MW	minimize $\|\Delta\text{MW}\|$	3.8040	3.9290	-0.1251 ± 3.6787	0.144	ns
		QED	maximize ΔQED	0.1001	0.0945	0.0056 ± 0.0313	1.86×10^{-8}	Original
E	793	QED	minimize QED drop	0.0328	0.0361	-0.0033 ± 0.0242	6.23×10^{-5}	Original
		SA	maximize $-\Delta$SA	0.4421	0.4314	0.0107 ± 0.1523	0.0244	Original
F	793	QED	minimize QED drop	0.0345	0.0329	0.0015 ± 0.0313	<u>0.0823</u>	<u>Ablation</u>
		SA	maximize $-\Delta$SA	0.7233	0.6903	0.0330 ± 0.1598	4.42×10^{-9}	Original

Supplementary Table 3: Summary statistics for the ablation study in Supplementary Figure 4. Original and ablation columns report objective-aligned Δ values averaged over paired input molecules. Paired differences are computed as original minus ablation (mean ± SD). For minimization objectives, negative paired differences favor the original model; for maximization objectives, positive paired differences favor the original model. P-values are obtained from one-sided paired t-tests. By default, the alternative hypothesis tests whether the original model outperforms the ablation. For underlined entries, the direction of the alternative hypothesis is reversed to test whether the ablation outperforms the original; therefore, smaller P-values in these cases indicate stronger evidence in favor of the ablation model. The "Preferred" column reports the model favored by the statistical test, with significance thresholds defined as ns $P > 0.1$, *$P < 0.1$, **$P < 0.05$, ***$P < 0.01$, and ****$P < 0.001$.

5.2 Analysis with additional test sets

In section 2.2 in the main manuscript, we constructed distinct test molecule sets for each property control scenario with different filtering criteria. They confined the test molecules to be appropriate for one property but not enough for the other property, intended to assume more practical situations. Thus, property control conditions were set to maintain the former and improve the latter. Here, for more statistical aspects of DeepBioisostere, we report results with randomly selected test molecules from ChEMBL that were not filtered in terms of properties that are optimized in each scenario. Still, the common filters in section 2.2 were applied: (1) a single neutral molecule, (2) molecular weight that falls within the range of 100 to 500, and (3) inclusion of at least one BRICS bond. 1,000 test molecules were randomly selected from the ChEMBL

database and used as original molecules to the DeepBioisostere model. We here compare property control results for this additional test set with the results for further filtered test sets in main manuscript Table 1, section 2.2.

Table 4 shows the results for three multi-property control scenarios with the test set constructed above. In scenario 2, where MW is to be kept and QED is improved, the avearge ΔMW is less than 1.0, same with that in main manuscript Table 1. However, QED improvements of 0.038 and 0.060 are much smaller than those in main manuscript of 0.067 and 0.100, respectively. This is due to that test molecules in section 2.2 are ones of which QED values are less than 0.4 so that they contain more room for QED improvement. Scenario 3 is the case where a given molecule's SAscore is improved while preventing its QED from decreasing. Compared to section 2.2 in the main manuscript, average ΔSAscore values of -0.209 and -0.285 are much weaker and average ΔQED values of -0.003 and +0.030 mean that QED was less degraded. Also in this scenario, since the given original molecules have worse SAscore for the Table 1 test set, they have more potential to be modified for better SAscore. These comparisons suggest that for the given property control condition and unsatisfying molecular structure, DeepBioisostere can find problematic sub-structures and generate appropriate bioisosteric replacements to optimize the target properties.

**Scenario**	**Requested Optimization**		**Property Change**	
	Property	ΔValue	Average	Std. Dev.
Scenario 1	MW	0	-0.19	12.7
	log$P$	+1	+0.84	0.35
	MW	0	-0.18	12.5
	log$P$	-1	-0.87	0.39
Scenario 2	MW	0	-0.20	10.6
	QED	+0.1	+0.038	0.091
	MW	0	-0.85	10.6
	QED	+0.2	+0.060	0.101
Scenario 3	QED	0	-0.003	0.099
	SAscore	-0.5	-0.209	0.262
	QED	0	+0.030	0.119
	SAscore	-1.0	-0.285	0.381

Supplementary Table 4: Results of three multi-property control scenarios with an additional test set. The requested optimization is the property control condition given to our model. For each scenario, we have two properties to control: Scenario 1 - modulating log$P$ while maintaining MW. Scenario 2 - increasing QED while keeping the molecular weight. Scenario 3 - decreasing SAscore without losing QED. ΔValue is the requested property change in each property. Average and Std. Dev. designates the mean value of property change and its standard deviation, respectively.

5.3 More statistical analysis on multi-property control scenarios

Supplementary Table 5: **Paired *t*-test results for property control scenarios.** For each scenario, both the maintained and target properties are listed with their mean Δ, standard deviation, *t*-statistics, and *p*-values. Two-tailed tests were applied to the maintained properties to verify that no significant deviation occurred, while one-tailed (directional) tests were used for the target properties to confirm whether their changes were statistically consistent with the intended direction of optimization.

Scenario	Condition	Maintain	Δ (mean ± SD)	t (vs 0)	p (vs 0)	Target	Δ (mean ± SD)	t (vs 0)	p (vs 0)	t (vs target)	p (vs target)
1	ΔMw 0 / ΔlogP +1	Mw	0.045 ± 6.53	0.22	0.83	logP	+0.831 ± 0.204	128.8	$< 10^{-60}$	-26.19	2.26×10^{-115}
1	ΔMw 0 / ΔlogP -1	Mw	-0.27 ± 6.19	-1.39	0.17	logP	-0.841 ± 0.217	-122.3	$< 10^{-60}$	+23.20	1.68×10^{-95}
2	ΔMw 0 / ΔQED +0.10	Mw	-0.13 ± 5.23	-0.78	0.44	QED	+0.067 ± 0.053	39.79	5.4×10^{-207}	-19.43	2.20×10^{-71}
2	ΔMw 0 / ΔQED +0.20	Mw	-0.58 ± 5.36	-3.37	7.8×10^{-4}	QED	+0.100 ± 0.062	49.98	7.6×10^{-272}	-50.34	9.29×10^{-274}
3	ΔQED 0 / ΔSA -0.50	QED	-0.026 ± 0.043	-17.01	1.4×10^{-55}	SA	-0.438 ± 0.361	-34.14	4.2×10^{-158}	+4.87	1.35×10^{-6}
3	ΔQED 0 / ΔSA -1.00	QED	-0.028 ± 0.045	-17.62	6.6×10^{-59}	SA	-0.719 ± 0.445	-45.42	5.3×10^{-223}	+17.77	1.01×10^{-59}

Table 5 summarizes the results of paired t-tests conducted for all property control scenarios and conditions described in the main manuscript. The analysis was performed using the same generated molecules presented in Table 1 of the main text, without additional sampling. To account for the stochastic nature of the generation process, each original molecule (e.g., n = 995, 979, 793 for Scenarios 1–3, respectively) had ten candidate structures generated, and the average property change across these variants was used as a single paired observation for the t-test. For each case, we evaluated whether (i) the mean change in the target property was significantly different from zero and (ii) whether it was statistically consistent with the requested numeric target. For the main property-control experiments, the mean shifts in the target properties were highly significant ($p \ll 0.001$), confirming that DeepBioisostere successfully modulates molecular properties in the desired direction. However, equivalence tests against the exact numeric targets (H_0: μ = target) were rejected in all settings, indicating that while directional control was clearly achieved, the improvements typically reached 50–88 % of the requested magnitude. This underachievement is expected since the model performs structure-preserving molecular modifications rather than de novo generation, thereby being constrained by the original molecular scaffolds and interdependencies among multiple properties. Nonetheless, the results demonstrate a clear monotonic relationship between the requested target magnitude and the realized degree of property change, validating the controllability and robustness of DeepBioisostere under realistic structural constraints.

6 Suppl. Note 6. Generated molecule examples and analysis

6.1 Property distributions of multi-property controlled molecules

DeepBioisostere demonstrated satisfactory performance in various multi-property control tasks. Specifically, in the first scenario aimed at increasing or decreasing $\log P$ while maintaining MW, the deviations of MW between generated molecules and the originals were 6.54 and 3.41, respectively. These results suggest that most replacements suggested by our model successfully maintained the original molecules' MW since the values are smaller than the MW of a single heavy atom.

To explore this further, we performed a statistical analysis of the generated molecules, focusing on their property deviation distributions. In Fig. 5(A), where the property control condition is *increasing* $\log P$ while retaining MW, most of the molecules are distributed between -20 and 20 for the MW deviation distribution. Likewise, Fig. 5(B), with the condition changed to *decreasing* $\log P$ while retaining MW, showed similar results, indicating that the generated molecules tend to have negligible change in MW.

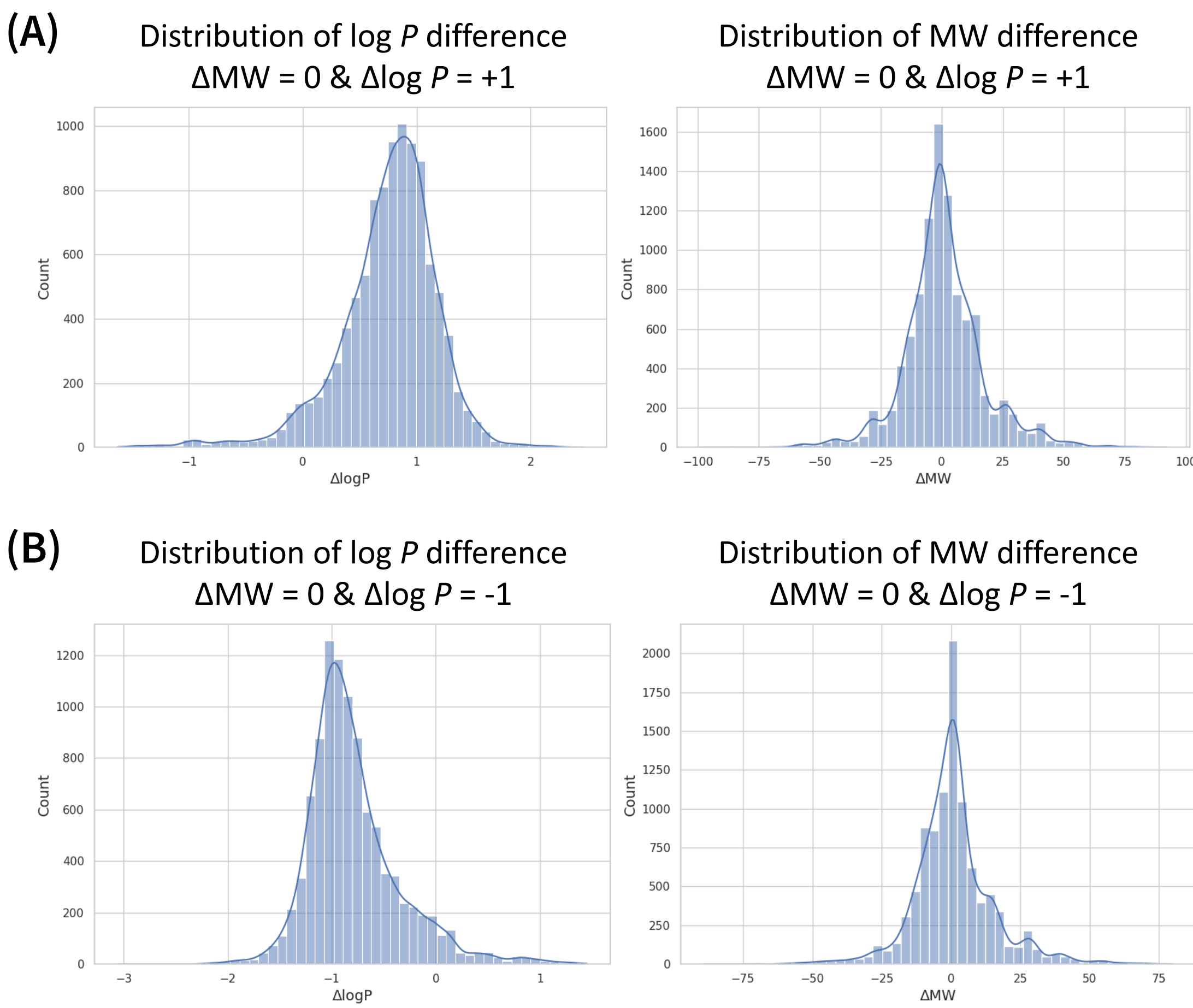

Supplementary Figure 5: Distributions of $\log P$ (left) and MW (right) deviations, computed from molecules generated by DeepBioisostere under the condition of increasing (A) or decreasing (B) $\log P$ by 1 while maintaining MW, alongside the original molecules.

6.2 More examples of bioisosteres for an uncommon fragment

In section 2.4 in the main text, we selectively exhibited the bioisosteres since many top-ranked fragments are structurally similar to each other. Here, for clarity, we provide 50 top-ranked fragments selected as a bioisostere for the azaindole moiety to maintain the QED value of the CHEMBL3403020 molecule. Most of them contain two aromatic rings, either fused rings or covalently bonded rings.

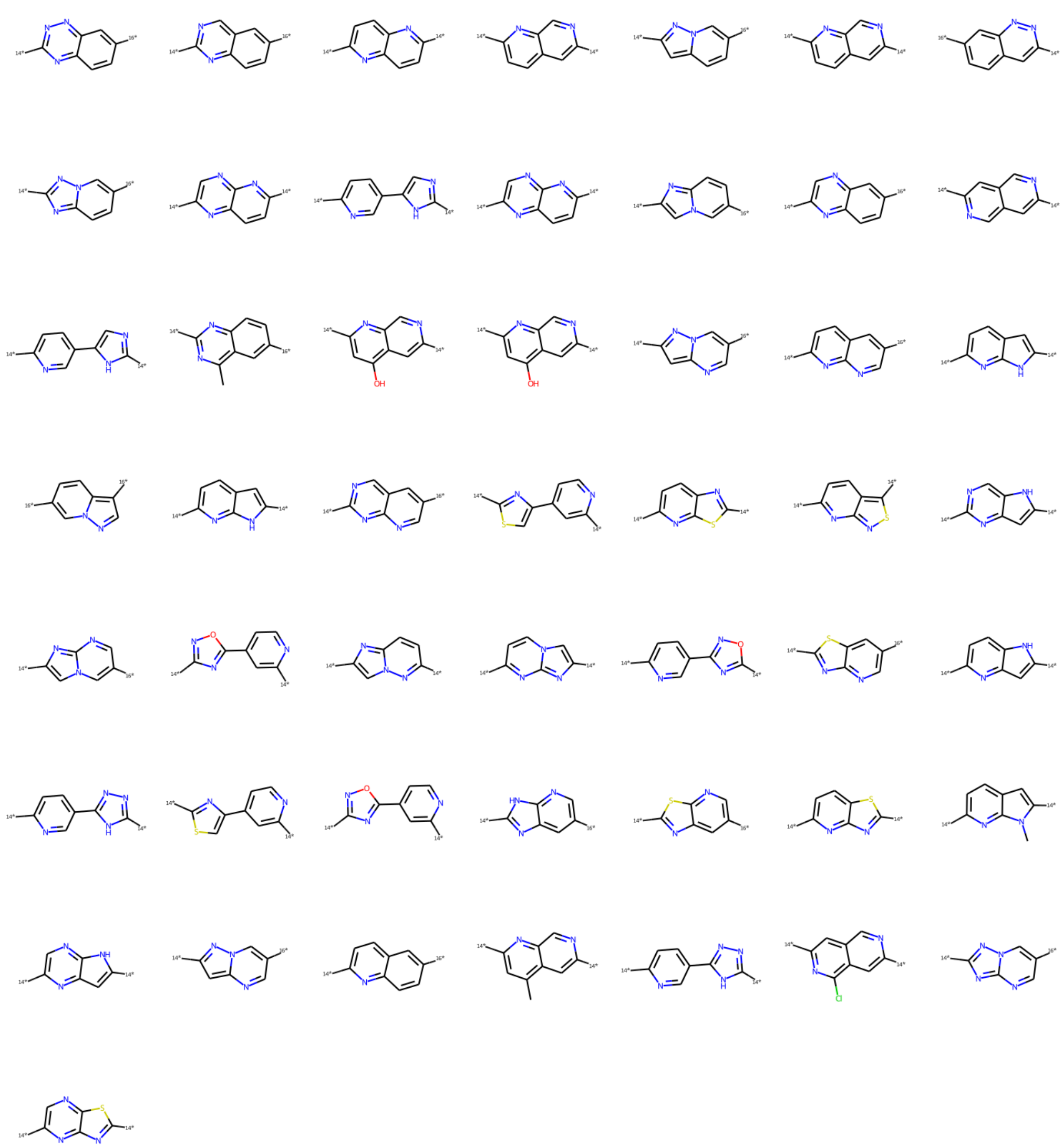

Supplementary Figure 6: Top 50 molecular fragments selected as the bioisosteres of an azaindole moiety in the molecule, CHEMBL3403020. Some duplicates exist since their different attachments to the remaining part result in different molecular structures.

7 Suppl. Note 7. Details on the *in silico* hit-to-lead workflow

7.1 Used structure-based 3D molecular generative models

In this section, four state-of-the-art models are adopted that are designed to generate 3D molecules for a given protein pocket. Pocket2Mol[3] and DeepICL[4] are auto-regressive models with a distinctive generation mechanism. Pocket2Mol sequentially adds atoms and bonds to generate a 3D molecular graph within a protein pocket. DeepICL sequentially adds atoms, explicitly considering non-covalent interactions with surrounding protein atoms. TargetDiff[5] and DecompDiff[6] are diffusion models, where TargetDiff denoises atom positions and types with a 3D equivariant diffusion model, while DecompDiff decomposes each ligand into a scaffold and arms and uses specialized priors for sampling.

7.2 Target selection criteria and statistics of generated molecules

With these four models, we first generate 100 molecules, each for 100 protein pockets from the test set of the CrossDocked2020 dataset,[7] a widely used benchmark for modern structure-based molecular generative models, following the protocol of Luo et al.[8] After generation, we computed the QED and SA score of each generated molecule. Then, we selected targets with an average QED of generated molecules lower than 0.6, and an average SA greater than 3.5, leading to three targets with PDB ID 4P77, 4RV4, and 2EWY, respectively. We provide the statistics of the average QED and SA score of the molecules generated by each model in Table 6.

**PDB ID**	**Model**	**QED (↑)**	**SA (↓)**
4P77	DeepICL	0.44	3.63
	Pocket2Mol	0.52	3.58
	TargetDiff	0.35	4.21
	DecompDiff	0.57	3.70
4RV4	DeepICL	0.44	3.88
	Pocket2Mol	0.57	3.51
	TargetDiff	0.24	4.85
	DecompDiff	0.39	4.06
2EWY	DeepICL	0.55	4.14
	Pocket2Mol	0.41	4.92
	TargetDiff	0.33	5.64
	DecompDiff	0.20	5.83

Supplementary Table 6: A table summarizing the average QED and SA scores for four different generative models across three selected targets.

7.3 Additional results

In Table 7, we provide additional results for the targets that are not shown in the main text.

PDB ID	Model	Strategy	Success Rate			
			QED	SA	Docking	Joint
4P77	DeepICL	Random	0.79	0.44	0.76	0.27
		Frequency-based	**0.82**	0.56	**0.85**	0.38
		MMPA-based	0.81	0.52	**0.85**	0.36
		DeepBioisostere	0.80	**0.63**	0.83	**0.44**
	Pocket2Mol	Random	**0.77**	0.45	0.66	0.22
		Frequency-based	0.76	0.51	**0.75**	0.28
		MMPA-based	**0.77**	0.44	**0.75**	0.23
		DeepBioisostere	0.74	**0.61**	0.68	**0.36**
	TargetDiff	Random	0.63	0.62	0.65	0.30
		Frequency-based	0.69	0.66	0.73	0.33
		MMPA-based	0.64	0.57	0.68	0.22
		DeepBioisostere	**0.83**	**0.75**	**0.74**	**0.49**
	DecompDiff	Random	0.25	0.30	0.58	0.05
		Frequency-based	0.46	0.42	0.83	0.15
		MMPA-based	0.43	0.40	**0.87**	0.16
		DeepBioisostere	**0.71**	**0.93**	0.82	**0.55**
2EWY	DeepICL	Random	0.15	0.10	0.95	0.01
		Frequency-based	0.24	0.20	**0.98**	0.05
		MMPA-based	0.48	0.15	0.95	0.06
		DeepBioisostere	**0.58**	**0.79**	0.90	**0.45**
	Pocket2Mol	Random	0.15	0.11	0.85	0.02
		Frequency-based	0.28	0.27	**0.89**	0.07
		MMPA-based	0.43	0.31	**0.89**	0.12
		DeepBioisostere	**0.63**	**0.75**	0.75	**0.34**
	TargetDiff	Random	0.13	0.30	0.93	0.05
		Frequency-based	0.24	0.44	**0.95**	0.10
		MMPA-based	0.30	0.36	**0.95**	0.10
		DeepBioisostere	**0.73**	**0.87**	0.92	**0.63**
	DecompDiff	Random	0.11	0.28	0.94	0.04
		Frequency-based	0.24	0.43	0.95	0.10
		MMPA-based	0.32	0.37	**0.96**	0.13
		DeepBioisostere	**0.76**	**0.92**	0.94	**0.69**

Supplementary Table 7: Additional results for two targets in the CrossDocked2020 benchmark dataset (PDB ID: 4P77 and 2EWY). The best success rates among replacement strategies are highlighted in bold.

Supplementary references

References

[1] Seo, S., Lim, J. & Kim, W. Y. Molecular generative model via retrosynthetically prepared chemical building block assembly. *Adv. Sci.* **10**, 2206674 (2023).

[2] Paszke, A. et al. PyTorch: an imperative style, high-performance deep learning library. *Adv. Neural Inf. Process. Syst.* **32**, 8026–8037 (2019).

[3] Peng, X. et al. Pocket2Mol: efficient molecular sampling based on 3D protein pockets. *Proc. Mach. Learn. Res.* **162**, 17644–17655 (2022).

[4] Zhung, W., Kim, H. & Kim, W. Y. 3D molecular generative framework for interaction-guided drug design. *Nat. Commun.* **15**, 2688 (2024).

[5] Guan, J. et al. 3D equivariant diffusion for target-aware molecule generation and affinity prediction. *International Conference on Learning Representations* (2023).

[6] Guan, J. et al. DecompDiff: diffusion models with decomposed priors for structure-based drug design. *Proc. Mach. Learn. Res.* **202**, 11827–11846 (2023).

[7] Francoeur, P. G. et al. Three-dimensional convolutional neural networks and a cross-docked data set for structure-based drug design. *J. Chem. Inf. Model.* **60**, 4200–4215 (2020).

[8] Luo, S., Guan, J., Ma, J. & Peng, J. A 3D generative model for structure-based drug design. *Adv. Neural Inf. Process. Syst.* **34**, 6229–6239 (2021).
