# Supplementary material for: Autonomous bioisosteric replacement for multi-property optimization in drug design
Source: Nat Commun. 2026 Jul 27;17:9113. doi: 10.1038/s41467-026-75512-9 (PMC13518832; doi:10.1038/s41467-026-75512-9)
Supplement: Supplementary file 2 [file 41467_2026_75512_MOESM2_ESM.pdf]

nature portfolio

Peer Review File

Autonomous Bioisosteric Replacement for Multi-Property Optimization in Drug Design

Corresponding Author: Professor Woo Youn Kim

This file contains all reviewer reports in order by version, followed by all author rebuttals in order by version.

Version 1:

Reviewer comments:

Reviewer #1

(Remarks to the Author)
The manuscript presents a method DeepBioisostere to do bioisosteric replacements through three steps: (1) removal fragment selection, (2) insertion fragment selection, and (3) attachment orientation prediction. DeepBioisostere is compared with a database mining method SwissBioisostere, a language-based method He et. al., and Modof, and is applied to optimize molecules for COVID19. The reviewer sees the following major issues that are required to be addressed:

1. Overall, the framework is highly similar to Modof. DeepBioisostere's step 1 is highly similar to the disconnection site prediction in Modof, step 2 and step 3 are very similar to Modof decoder, which samples a latent vector and predicts using the vector where to add new atoms/bonds and when to stop. Also, AMPN and FMPN are highly similar to the GMPN and TMPN as in Modof, respectively – AMPN and GMPN are identical in their ideas; FMPN and TMPN are highly similar, with FMPN using BRICS based structures, and TMPN uses junction-tree based structures, but idea-wise they are the same idea; also, the idea of two graphs: atom + fragment, is the same as Modof. The manuscript is presented in a way that AMPN and GMPN are new, but they are not and are the same as in Modof. The authors very briefly mentioned "Largely inspired by Chen et al. [35]" in 4.3.1, however, the high similarity in the frameworks and ideas to Modof is not clearly articulated and attributed to Modof. The similarity to Modof needs to be clearly described in the manuscript. Any claimed novelty needs to be clearly distinguished from the existing methods with clear citation/explicit referral to the existing methods; if methods use the same ideas with existing methods but are rephrased using different names or with minor changes (e.g., atom features, fragmentation methods), they should clearly attribute to the original methods whenever these methods are described.

2. The test set with 110 molecules is way too small to give statistically significant results – all the test sets in related research topics (e.g., molecule generation, molecule optimization, binding affinity prediction) are much larger than 110 (e.g., > 10x). It is not clear why QED > 0.8 is used to construct the test set. QED threshold can only determine how hard QED improvement can be (how much QED improvement is bounded), but does not prevent the methods from showing their abilities to improve QED – for example, for low QED, the improvement can be higher. The authors need to use a larger test set – at least close to 1,000 testing cases.

3. Also related to statistical significance in the results, in Table 2, results in scenarios 2 and 3 do not appear significant. For example, in scenario 2, the goal is to improve QED by 0.2, but the results have QED improvement on average 0.087, which is far below 0.2; meanwhile, the standard deviation is high – 0.101. Two statistical significance tests are needed: (1) whether the improvement is significantly above 0; and (2) whether the improvement is significantly different from the requirement. Confidence intervals should also be presented. Also, in supplementary Figures 2 and 6, statistical testing is needed to test if delta is significantly different from 0. In general, whenever there is a claim about difference, there should be significance test (e.g., for Fig 3(b)). It appears that all the standard deviations in the results are very high (Table 1, Table 2). Given the test set is so small, this is highly concerning.

4. Regarding the comparison with Modof in Table 1: Modof is trained to optimize plogP, which is a combination of logP, molecule complexity and synthesizability (SAscore). While DeepBioisostere is trained to optimize logP, it is unfair to compare the plogP-optimized Modof with DeepBioisostere on logP. In addition, DeepBioisostere's training data is very different from the benchmark data for molecule optimization that Modof uses, and thus, there will be significant distribution shift issue for Modof. Given that DeepBioisostere's training data is much larger than Modof's, the authors need to train Modof using DeepBioisostere's training data and DeepBioisostere's objective, that is, to improve logP, not Modof's original

objective to improve plogP, and then compare DeepBioisostere and Modof. The authors need to have fair comparisons among all the methods including SwissBioisostere and He et.al., making sure all the methods are optimized with respective to the same property and use same training data; it is unfair to compare methods that are not optimized for DeepBioisostere's objective and claim they are worse than DeepBioisostere with respective to DeepBioisostere's objective.

5. The pre-trained model of DeepBioisostere with all the model parameters and hyperparameters, as well as the processed training and test data, need to be published. The authors only published the code to process data and train models. Also, the new molecules with fragments replaced should also be published.

6. There are quite a few multi-property optimization papers. In addition, though not specifically designed, Modof model can also be adapted to multi-property optimization tasks by altering its objective. However, this paper does not discuss them in the related work or compare with them as baselines. Below are examples for multi-property optimization papers:
a. Jin, Wengong, Regina Barzilay, and Tommi Jaakkola. "Multi-objective molecule generation using interpretable substructures." International conference on machine learning. PMLR, 2020.
b. Fu, Tianfan, et al. "Mimosa: Multi-constraint molecule sampling for molecule optimization." Proceedings of the AAAI Conference on Artificial Intelligence. Vol. 35. No. 1. 2021.
c. Loeffler, Hannes H., et al. "Reinvent 4: Modern AI–driven generative molecule design." Journal of Cheminformatics 16.1 (2024): 20.
More comparison results with multi-property optimization methods are needed.

7. The authors claimed that DeepBioisostere is "a deep generative model for multi-property control with designing suitable bioisosteric replacements." However, DeepBioisostere essentially screens all the fragments in the pre-defined library in order to decide which fragment should be used as a replacement. This is not "generative" – it does not generate new fragments as Modof and many other generative models (e.g., JTNN) do; instead, it looks for and literally tries existing fragments. DeepBioisostere produces new molecules by replacing fragments; however, even in this sense, the notion of "generative" is much weaker than other molecule generation methods. Thus, "generative" is misleading and is a misconception to claim DeepBioisostere.

8. Section 2.4.2 appears very odd. First of all, the notations A->A and B->B are very confusing. Second, this exercise shows the importance of the chemical environment, which is general knowledge. It does not show whether DeepBioisostere can better leverage the environment, particularly automatically and in comparison with other methods. Also, figure 3(b) does not show a significant difference among all three cases in their delta logP; a significance test is needed.

9. In Discussion section, there are no discussions about the potential issues of DeepBioisostere.

(Remarks on code availability)
The pre-trained model of DeepBioisostere with all the model parameters and hyperparameters, as well as the processed training and test data, need to be published. The authors only published the code to process data and train models.

Reviewer #2

(Remarks to the Author)
The manuscript submitted by the Authors describes an interesting and novel way of finding bioisosteric replacements in small molecules. DeepBioisostere is a deep learning-based approach that includes three steps: identification of the fragment for replacement, selection of the new fragment, and prediction of the attachment points. The model is trained on a dataset of extracted matched molecular pairs (MMP) curated from ChEMBL. The problem of bioisosteric replacement is formulated as a conditional probability that can be factorized into three steps modeled by graph neural networks. These networks operate on the atom and fragment levels. The experiments show that DeepBioisostere can be used to optimize some molecular properties while retaining others. It also beats three other methods for molecular optimization using bioisosteric replacement. A case study shows that DeepBioisostere can be used to design drug analogs to combat drug resistance of one of the SARS-CoV-2 variants.

The manuscript is well-written, and the results are significant in the field of computational drug discovery. However, some design decisions could be explained in more detail, and the experiments could be improved. My detailed comments are included below.

1. I am curious about the Authors' definition of bioisosteres. I learned that bioisosteric replacements, as suggested by the name, are usually very subtle changes that do not change the shape or electronic properties of a compound so that it can preserve its bioactivity while optimizing other properties. The replacements proposed by DeepBioisostere are often more extensive and change the overall shape of the molecule. Would it not be considered scaffold hopping?

2. Following the logic in the previous point, demonstrating that DeepBioisostere can modify the molecular structure to optimize, e.g., solubility without changing the binding pose and predicted affinity would be an interesting case study that would prove the method useful in a more classical application of bioisosterism.

3. The motivation of the paper is clear. The automatic selection of the fragment for replacement is important for streamlining the lead optimization process. However, one sentence in the introduction is difficult to understand: "Also, the expectation of property changes based on the mined data may not work for more complicated properties that depend on complex relationships between internal moieties, such as synthesizability or drug-likeness." Could you please rephrase this

sentence?

4. Most of the related work is described in the introduction, which puts this research in the context of other methods for molecular optimization and bioisosteric replacement. I recommend including a few articles about machine learning-based scaffold hopping, which present a fully automated procedure of molecular optimization. It would be interesting to see a few methods of this kind in the experiment presented in Table 1 as an example of different approach to molecular optimization. For example:
[1] Zheng, S., Lei, Z., Ai, H., Chen, H., Deng, D., & Yang, Y. (2021). Deep scaffold hopping with multimodal transformer neural networks. Journal of cheminformatics, 13, 1-15.
[2] Hu, C., Li, S., Yang, C., Chen, J., Xiong, Y., Fan, G., ... & Hong, L. (2023). ScaffoldGVAE: scaffold generation and hopping of drug molecules via a variational autoencoder based on multi-view graph neural networks. Journal of Cheminformatics, 15(1), 91.

5. While the approach described in the manuscript appears to be novel, I do not think the following sentence is true: “Note that DeepBioisostere is the first generative approach to the bioisosteric replacement for molecular optimization.” There are already at least a few examples of other generative models that can perform bioisosteric replacement, e.g., Shan and Ji [3] generate SMIRKS rules with recurrent neural networks, and Maziarka et al. [4] formulate bioisosteric replacement as a domain adaptation task. These approaches are significantly different, but nonetheless, they are examples of generative models for bioisosteric replacement.
[3] Shan, J., & Ji, C. (2020). MolOpt: a web server for drug design using bioisosteric transformation. Current computer-aided drug design, 16(4), 460-466.
[4] Maziarka, Ł., Pocha, A., Kaczmarczyk, J., Rataj, K., Danel, T., & Warchoł, M. (2020). Mol-CycleGAN: a generative model for molecular optimization. Journal of Cheminformatics, 12(1), 2.

6. The results described in Sections 2.2 and 2.3 are impressive and compelling. However, only very simple properties are considered in these experiments. I know these are standard descriptors often used in the literature to evaluate machine learning models, but I wonder how easy it is to apply more advanced molecular properties here. If I understand correctly, these properties need to be known for all training molecules because they are a part of the conditional distribution p(M’|M,C). It is only possible because cLogP, QED, and SA can be computed in milliseconds. The task becomes much more difficult if someone wants to use wet-lab bioactivity data (virtually impossible to achieve for almost a million ChEMBL molecules) or even more advanced simulated data like molecular docking results. Applying machine learning models could be a solution, but training an ML model with an applicability domain as big as the whole ChEMBL dataset is still infeasible. If my understanding is correct, this needs to be described as a limitation of the proposed model. Could you suggest any solution to that problem? For example, can you train DeepBioisostere with partially labeled data?

7. All the figures in the manuscript are very informative, especially Figures 2 and 3, which provide readers with a better understanding of the inner workings of the proposed algorithm. I also enjoyed the case study on targeting the SARS-CoV-2 main protease mutant. This well-designed experiment showed an example of a real-life application even though only simple computed properties like cLogP could be optimized.

8. The architecture design of DeepBioisostere is well thought-out. I am curious about a few design decisions that seem nonstandard in the machine learning practice. The Authors can comment on these aspects.
a) Why is the a_i term inside the sum in Equation 2? This term is independent of a_j, so it can be moved outside the sum. Now, the signal from the central node is stronger, but it can be canceled by the trainable weights. However, it seems inefficient from the computation perspective.
b) Why is GRU used in Equation 3? Recurrent neural networks are typically used to process sequences. What is the intuition behind using GRU instead of a simple activation function?
c) Equation 5 treats the atoms inside the replaced fragment the same way as the atoms in the rest of the molecule (there is no direction of the bonds). Could adding information about the direction (inside vs outside the replaced fragment) help the model learn better representation?
d) A binary cross-entropy is used in the loss functon(Eq. 12). Why have you decided not to use a multi-class cross-entropy? What if two MMPs are found for a molecule, and two different fragments are positive samples?
e) A negative sign is typically used on the right-hand side of equations when defining loss functions (Eqs. 12-14).

9. I recommend including an ablation study in the manuscript that shows the proposed model's results when other architectures or sampling strategies (Section 4.4) are used. This experiment would explain the design decisions described above and provide a better understanding of the model.

In conclusion, the manuscript is well-written, and the proposed method seems innovative and practical. However, my main concerns are regarding the interpretation of bioisosteric replacement, especially in relation to some medicinal chemistry literature that describes bioisosteres as small changes that preserve the shape of a molecule. DeepBioisostere does not offer any control over the similarity of the inserted fragments or the maximum shape or electronic structure change of the output molecule. Additionally, applying the method to advanced properties is challenging. Extensive modifications of the input compound may impact multiple molecular properties, which cannot be easily controlled due to the reasons mentioned above. These limitations must be addressed or adequately described in the manuscript. Thus, I recommend a major revision.

(Remarks on code availability)

I managed to install the conda environment and run the code in the example notebook. Unfortunately, a trained model is not provided, and the data used for training is not available yet, so I could not test the generator. The usage is properly described in the README file, which includes model training and inference, but `jupyter notebook` needs to be downloaded to run the example in the ipynb file (this dependency is not included in the README file nor in the `dependencies` script). The code is organized in a clear structure. The code that reproduces plots in Figures 2, 3, and 4 is provided. I have not tried training the model from scratch because the training data is not provided, which makes the results in the paper irreproducible.

Reviewer #3

(Remarks to the Author)
The authors present a deep generative model that automatically replaces fragments in small molecules with the purpose of optimizing calculated properties, while keeping others unchanged, relevant for drug discovery. The authors stipulate that their method is generating bioisosteric replacements that go beyond what is known from bioisostere databases or the medicinal chemistry literature at large.

In the scientific literature and by practitioners at large, the term bioisosteric replacement is used to describe the replacement of a part of a bioactive molecule with another substructure of similar size and similar properties. The term is generally applied to replacements that have been observed experimentally exhibiting an effect of maintaining experimental properties consistently across multiple examples. For instance, Ertl requires at least five or more experimental examples used with limited spread in observed property differences (<1 log unit) before he is comfortable calling a replacement a bioisostere [ref 29]. The authors attempt to extend the concept of bioisosteres beyond experimentally observed property similarity data to calculated property data. As a result, the number of potential 'bioisosteric' replacements goes up significantly. In doing so, the authors leave the idea of experimental validation behind, extending the concept of bioisosteres instead to all matched molecular pairs (MMPs) of compounds with bioactivity <=10uM, regardless of experimentally validated property maintenance IN THE SAME ASSAY. In my opinion, this extension to calculated-only properties, without consideration of experimental data, is not useful as it softens the term bioisostere at best and almost completely deprives it of its meaning at worst. Rather, it extends the term bioisostere to that of MMPs (of compounds with any bioactivity against in at least one assay), which are not the same.

If I take the term 'bioisostere' and its underlying notion out of consideration for a moment, I see an algorithm and method that is not unlike other generative AI reinforcement models, such as REINVENT4, that generate compound ideas driven by a fitness function that can easily incorporate the parameters chosen by the authors of the manuscript, as they relate to logP, QED, MW, synthesizability score, etc. For this purpose, I do not see a particular advantage of limiting the generative AI to matched molecular pairs derived from Chembl. Therefore, I do not see a meaningful novelty demonstrated in the approach presented in the manuscript.

The author's claim of their method outperforming data mining approaches for bioisosteres is not supported by the data as the author's approach is not strictly searching for established bioisosteres but for hypothetical replacements that are not proven to be bioisosteres yet.
While the authors show that their method generated compounds with the desired calculated properties, the comparison to other approaches such as Swissbioisostere is not done at equal footing as Swissbioisostere includes statistical information on the impact of replacements on bioactivities, while the author's method does not seem to take such data into account.

The authors apply their method to identifying inhibitors of the 3CLpro E166V mutant. None of the suggested inhibitors have been synthesized and tested. The claims of improved properties of the putative inhibitors are not supported by experimental evidence.

For the above reasons, I believe that the manuscript is not suitable for publication in Nature Communications.

(Remarks on code availability)

Version 2:

Reviewer comments:

Reviewer #2

(Remarks to the Author)
I want to thank the Authors for addressing my comments. After reading the other reviews, responses, and the revised manuscript, I have only a few minor comments:

1. Supplementary Figure 4 does not clearly show the difference between the two distributions. The boxplots look very similar even when $P<0.001$ (because the paired t-test is used, even small but consistent differences between paired values may be statistically significant). Reporting, for instance, the mean difference in delta values between the original model and its ablation, along with the standard deviation, would help readers understand why the original model is significantly better than the ablated one.

2. On page 21, the meaning of C_i (with an index) should be clarified.

3. Typo in Section 5.1 of the Supplementary Information: "proceduer"

The manuscript introduces a useful computational method that can aid in discovering new drugs, especially during the lead optimization stage of drug design. The experiments demonstrate that DeepBioisostere can effectively optimize multiple molecular properties simultaneously. The code has been updated to ensure reproducibility of the results, and the model has been released under the MIT license. Based on these points, I recommend acceptance after minor revisions.

(Remarks on code availability)
The code repository has been updated and now includes model parameters and processed datasets.

Reviewer #3

(Remarks to the Author)
The authors adequately addressed my previous concerns.

(Remarks on code availability)

Open Access This Peer Review File is licensed under a Creative Commons Attribution 4.0 International License, which permits use, sharing, adaptation, distribution and reproduction in any medium or format, as long as you give appropriate credit to the original author(s) and the source, provide a link to the Creative Commons license, and indicate if changes were made.
In cases where reviewers are anonymous, credit should be given to 'Anonymous Referee' and the source.
The images or other third party material in this Peer Review File are included in the article's Creative Commons license, unless indicated otherwise in a credit line to the material. If material is not included in the article's Creative Commons license and your intended use is not permitted by statutory regulation or exceeds the permitted use, you will need to obtain permission directly from the copyright holder.
To view a copy of this license, visit https://creativecommons.org/licenses/by/4.0/

Dear Editor,

Please find the enclosed manuscript for your consideration to publish in *Nature Communications* as an article entitled "**Autonomous Bioisosteric Replacement for Multi-Property Optimization in Drug Design**", authored by Hyeongwoo Kim, Seokhyun Moon, Wonho Zhung, Shinwoo Kim, Jaechang Lim, and Woo Youn Kim.

We sincerely appreciate you and the reviewers for their thoughtful and constructive feedback on our previous submission Manuscript: ID **'NCOMMS-24-20570A-Z'**). The major concern involved the definition of bioisosterism used in our work which may cause an overly broad interpretation of bioisosteric replacements and thus raises doubts about whether the bioactivity of generated molecules can be experimentally validated. We fully acknowledged this issue and have undertaken substantial revisions in response.

In the revised manuscript, we systematically curated a new training dataset following the standard procedure of defining bioisosteres, ensuring consistency with conventional medicinal chemistry practices. Moreover, in the previous version of our manuscript, we included a benchmark comparing our method to traditional database-mining approaches and deep generative models for molecular property optimization, which served as baselines. However, following further reflection and in alignment with the reviewers' concerns that highlighted the fairness of the comparison, we decided to redesign the benchmark in the revised manuscript. The newly designed benchmark evaluates the bioactivity and property control of the molecules designed by recent molecular generative models for *de novo* drug design, which reflects a more realistic scenario where the bioisosteric replacement is required to improve both properties and synthesizability. This benchmark compares our method against standard bioisosteric replacement strategies and demonstrates a key advancement; our model can autonomously modulate multiple properties—such as drug-likeness and synthetic accessibility—while preserving bioactivity, by considering structural compatibility within the full molecular context.

Through this revision, we believe that the statistical reliability of our curated training dataset has been substantially enhanced, thereby reinforcing the robustness of our model. Furthermore, we incorporated additional analysis that better demonstrates the practical utility and versatility of *DeepBioisostere*. We are confident that the revised manuscript, together with the growing body of recent deep learning–based molecular design approaches, will contribute to advancing more realistic, data-driven strategies for *in silico* drug discovery.

Thank you for reconsidering our manuscript for publication. We eagerly anticipate further feedback and the opportunity to contribute to advancing scientific knowledge in your esteemed journal.

Sincerely yours,

Woo Youn Kim,

Professor of Chemistry, KAIST.

Point-by-point replies to the feedback are as follows.

<u>Reviewer 1.</u>

<u>Q1</u>. Overall, the framework is highly similar to Modof. DeepBioisostere's step 1 is highly similar to the disconnection site prediction in Modof, step 2 and step 3 are very similar to Modof decoder, which samples a latent vector and predicts using the vector where to add new atoms/bonds and when to stop. Also, AMPN and FMPN are highly similar to the GMPN and TMPN as in Modof, respectively – AMPN and GMPN are identical in their ideas; FMPN and TMPN are highly similar, with FMPN using BRICS based structures, and TMPN uses junction-tree based structures, but idea-wise they are the same idea; also, the idea of two graphs: atom + fragment, is the same as Modof. The manuscript is presented in a way that AMPN and GMPN are new, but they are not and are the same as in Modof. The authors very briefly mentioned "Largely inspired by Chen et al. [35]" in 4.3.1, however, the high similarity in the frameworks and ideas to Modof is not clearly articulated and attributed to Modof. The similarity to Modof needs to be clearly described in the manuscript. Any claimed novelty needs to be clearly distinguished from the existing methods with clear citation/explicit referral to the existing methods; if methods use the same ideas with existing methods but are rephrased using different names or with minor changes (e.g., atom features, fragmentation methods), they should clearly attribute to the original methods whenever these methods are described.

<u>Reply</u>: First of all, we sincerely thank the reviewer for this feedback. Indeed, as only briefly mentioned in the original manuscript, the architecture design of *DeepBioisostere* was largely inspired by *Modof*. For effective learning and inference of bioisosteric replacement in a fragment-wise decision-making process, it is essential to capture both the representation of molecular fragments and their relationships. Given the hierarchical modeling strategy proposed in *Modof*—consisting of GMPN and TMPN—and its strong performance on molecular optimization tasks, we expected that a similar architectural strategy would also be suitable for our goal. Accordingly, we adopted a strategy in which message passing is performed on the atom-level graph and then aggregated into a higher-level structure (junction-tree graph or fragment-level graph) for subsequent message passing.

Three important differences distinguish DeepBioisostere from Modof, which are tailored for the specific task of bioisosteric replacement:

1. **Direct Relational Modeling:** DeepBioisostere directly models the relational nature of bioisosteric replacement, the main goal of this work, whereas Modof does not. Modof learns separate latent spaces for encoding and decoding the fragment-to-remove and fragment-to-add, respectively. In contrast, DeepBioisostere explicitly learns the relationship between fragment pairs: the insertion module is directly conditioned on a concatenated embedding of both the removal fragment and the candidate insertion fragment. This contrastive design explicitly evaluates the "fit" of one fragment for another, which is central to bioisosterism.
2. **Generative Strategy (Decoding vs. Retrieval):** The two models employ different generative strategies for structural modification. Modof generates a new fragment by decoding it from a latent vector, sequentially predicting new child nodes and

attachment points. In contrast, DeepBioisostere defines a probability distribution over a large, predefined fragment library and then retrieves the most suitable insertion fragment, which is an essential process for bioisosteric replacement. The subgraph AMPN module is critical for this retrieval-based strategy, as it provides a consistent embedding space that enables the model to reliably compare candidate fragments and select the best replacement.

3. **Training dataset for bioisosterism**: DeepBioisostere is trained on a dedicated bioisosteric replacement dataset that we constructed specifically to enable target-agnostic bioactivity control, whereas Modof is not. The training dataset consists of molecular pairs drawn from the same bioassays that are experimentally observed to exhibit similar bioactivities, thereby statistically defining bioisosteric fragment pairs. In addition, the training dataset includes only replacements permitted by the BRICS rules, favoring retrosynthetically accessible structural modifications and contrasting with Modof's junction-tree-based bond disconnection scheme.

As the reviewer correctly pointed out, the original manuscript did not sufficiently clarify which parts of DeepBioisostere were adopted from existing methods (e.g., *Modof*) and which parts represent our unique contributions. We fully acknowledge this issue and have revised Section 4.2.1 of the manuscript to explicitly describe:

(i) which architectural designs are directly adapted from *Modof*,
(ii) which are newly designed in *DeepBioisostere*, and
(iii) how they are combined to meet the requirements of the molecular property control task.

The revised a paragraph in Section 4.2.1 is as follows:

"We adopted the core architectural design of Modof proposed by Chen et al. [38], which demonstrates strong molecular property conditioning performance through a hierarchical framework. In its design, as illustrated in Fig. 7, atom-wise embed- dings are first generated using an atom-level graph message passing network, then aggregated into fragment-level hidden vectors, which are subsequently processed by a fragment-level graph message passing network. In DeepBioisostere, these two message passing networks are adapted for bioisosteric replacement and are referred to as the atom message passing network (AMPN) and fragment message passing network (FMPN), respectively. To tailor the model for bioisosteric replacement, we design the AMPN with two submodules having distinct parameters: a whole-graph AMPN operating on all molecular nodes and a subgraph AMPN operating only within individual fragments. The subgraph AMPN is employed as a shared fragment embedder for both the original molecule and the fragment library, providing a consistent and comparable representation of molecular fragments. This weight-sharing strategy ensures a comprehensive understanding of fragments and, in turn, allows the overall generative model to be formulated as a product of fragment-level conditional probabilities (Eq. (1)). In contrast to Modof, which decodes new fragments independently from separate latent vectors, DeepBioisostere evaluates candidate replacements by directly comparing the joint embedding of the removal fragment and each candidate insertion fragment from its library in this shared space. Furthermore, when combined with our same-bioassay molecular pair dataset, this molecular encoding strategy enables the model to capture similarity in bioactivity as well as differences in other molecular properties. We

quantitatively analyze the contribution of this architectural design to multi-property optimization in Supplementary Information Section 5.1."

In order to further validate the utility of our architectural design, we additionally performed an ablation study on the usage of the subgraph AMPN as a shared fragment embedder, which demonstrates that the unique components of DeepBioisostere indeed confer advantages for multiple molecular property control. The ablation study shows that the subgraph AMPN contributes to superior multi-property control performance across all scenarios in the manuscript with high significance level. Detailed analysis is provided in Supporting Information section 5.1, and the corresponding t-test results are presented in SI Fig. 4:

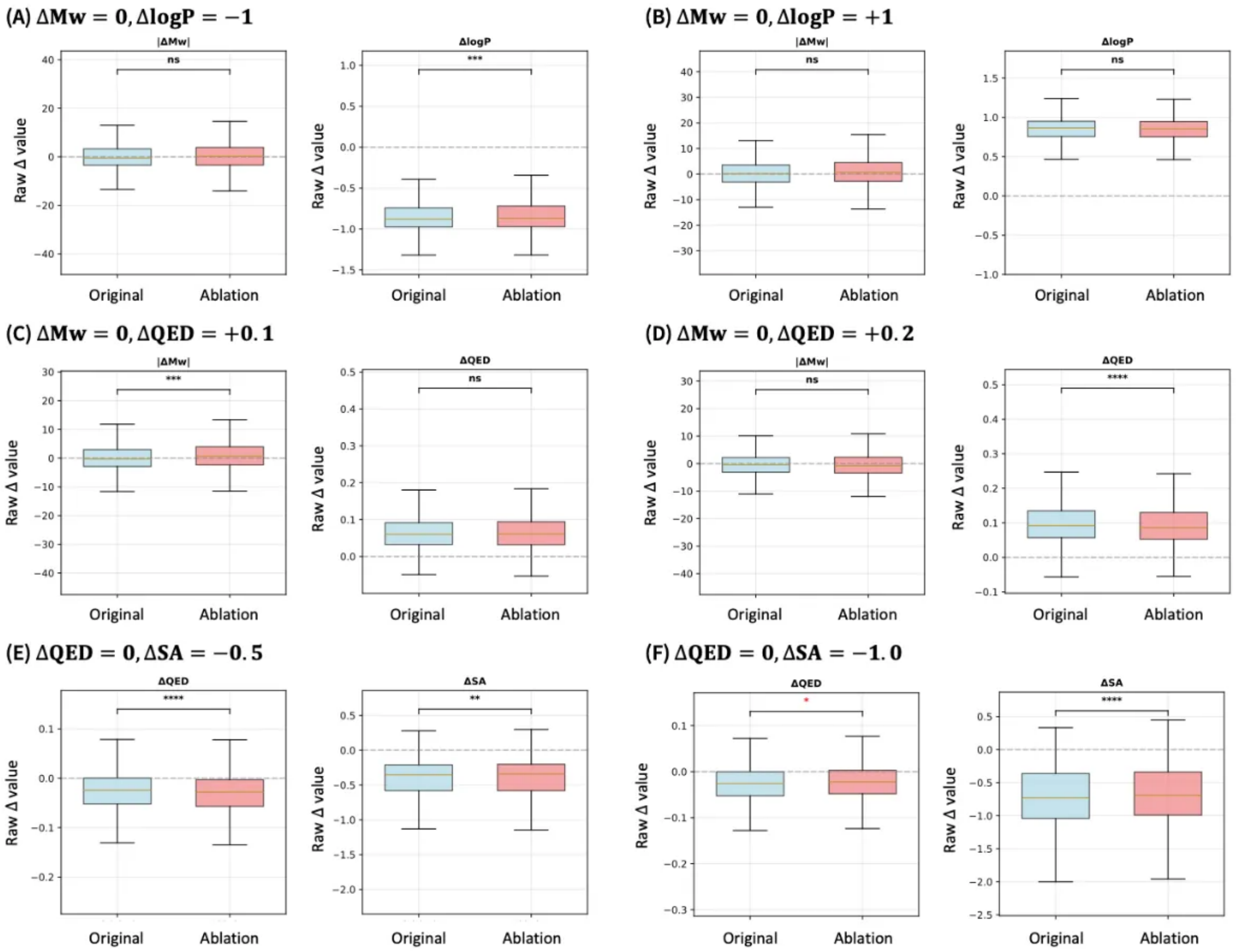

Supplementary Figure 4: Paired t-test results to compare multi-property control performances; ns $P > 0.1$, *$P < 0.1$, **$P < 0.05$, ***$P < 0.01$, and ****$P < 0.001$. Black stars designate the original model statistically achieves higher performance, and red ones mean the ablated model exhibits better results. Used input property control conditions are shown in sub-titles. (A,B) Scenario where MW and $\log P$ are modulated simultaneously. (C,D) Scenario where MW and QED are modulated simultaneously. (E,F) Scenario where QED and SA are modulated simultaneously.

<u>Q2</u>. The test set with 110 molecules is way too small to give statistically significant results – all the test sets in related research topics (e.g., molecule generation, molecule optimization, binding affinity prediction) are much larger than 110 (e.g., > 10x). It is not clear why QED > 0.8 is used to construct the test set. QED threshold can only determine how hard QED improvement can be (how much QED improvement is bounded), but does not prevent the methods from showing their abilities to improve QED – for example, for low QED, the improvement can be higher. The authors need to use a larger test set – at least close to 1,000 testing cases.

<u>Reply</u>: We fully agree with the reviewer's concern. The test set of 110 molecules is too small to ensure statistical confidence, and we acknowledge that the rationale for using the QED > 0.8 threshold was unclear. To address these issues, we performed two additional experiments:

1. **Larger test set with relaxed QED threshold.**

   We relaxed the QED threshold to > 0.7, which allowed us to collect a larger pool of candidate molecules. After applying the same similarity filtering to exclude overlaps with training data, we randomly sampled 1,000 test molecules. This enabled us to substantially expand the test set and improve statistical reliability. ****We have revised Table 1 accordingly:

Scenario	Requested Optimization		Property Change	
	Property	ΔValue	Average	Std. Dev.
Scenario 1	MW	0	+0.04	12.8
	log$P$	+1	+0.83	0.38
	MW	0	-0.28	12.6
	log$P$	-1	-0.84	0.43
Scenario 2	MW	0	-0.11	12.1
	QED	+0.1	+0.067	0.090
	MW	0	-0.57	12.0
	QED	+0.2	+0.100	0.099
Scenario 3	QED	0	-0.026	0.073
	SAscore	-0.5	-0.424	0.433
	QED	0	-0.028	0.074
	SAscore	-1.0	-0.719	0.524

Table 1: **Results of three multi-property control scenarios.** The requested optimization is the property control condition given to our model. For each scenario, we have two properties to control: Scenario 1 - modulating log$P$ while maintaining MW. Scenario 2 - increasing QED while keeping the molecular weight. Scenario 3 - decreasing SAscore without losing QED. ΔValue is the requested property change in each property. Average and Std. Dev. designates the mean value of property change and its standard deviation, respectively.

2. **Unrestricted QED evaluation.**

   Following the reviewer's suggestion, we also constructed a test set for all scenarios in Table 1 without any property restriction, randomly selecting molecules for evaluation. The results for this test set are reported in Supplementary Information Table S3 and can be summarized as follows:

Scenario	Requested Optimization		Property Change	
	Property	ΔValue	Average	Std. Dev.
Scenario 1	MW	0	-0.19	12.7
	log$P$	+1	+0.84	0.35
	MW	0	-0.18	12.5
	log$P$	-1	-0.87	0.39
Scenario 2	MW	0	-0.20	10.6
	QED	+0.1	+0.038	0.091
	MW	0	-0.85	10.6
	QED	+0.2	+0.060	0.101
Scenario 3	QED	0	-0.003	0.099
	SAscore	-0.5	-0.209	0.262
	QED	0	+0.030	0.119
	SAscore	-1.0	-0.285	0.381

Supplementary Table 3: **Results of three multi-property control scenarios with an additional test set.** The requested optimization is the property control condition given to our model. For each scenario, we have two properties to control: Scenario 1 - modulating log$P$ while maintaining MW. Scenario 2 - increasing QED while keeping the molecular weight. Scenario 3 - decreasing SAscore without losing QED. ΔValue is the requested property change in each property. Average and Std. Dev. designates the mean value of property change and its standard deviation, respectively.

When compared to Table 1 of the main manuscript, Scenario 1 shows similar results, whereas Scenarios 2 and 3 exhibit clear differences. Specifically, in Scenarios 2 and 3, property preservation improved, while the degree of property improvement was smaller. This is consistent with our test set design: in Table 1, we intentionally selected molecules that already had well-developed values for the property to be maintained, while the property to be improved was underdeveloped.

Regarding the concern about the QED threshold, we would like to clarify a potential misunderstanding in our original manuscript. The reviewer rightly pointed out that "for low QED, the improvement can be higher." We completely agree, and this was precisely our rationale for Scenario 2 (QED improvement). In this scenario, as stated in Section 2.2, we specifically used molecules with QED < 0.4 to demonstrate a meaningful improvement. The high QED threshold (from the original > 0.8 to > 0.7) was used for a different task, Scenario 3, where the goal was not to improve QED, but rather to improve the SAscore while maintaining the already high enough QED. We hope the new experiments and this explanation fully address your concerns.

<u>Q3</u>. Also related to statistical significance in the results, in Table 2, results in scenarios 2 and 3 do not appear significant. For example, in scenario 2, the goal is to improve QED by 0.2, but the results have QED improvement on average 0.087, which is far below 0.2; meanwhile, the standard deviation is high – 0.101. Two statistical significance tests are needed: (1) whether the improvement is significantly above 0; and (2) whether the improvement is significantly different from the requirement. Confidence intervals should also be presented. Additionally, in Supplementary Figures 2 and 6, statistical testing is required to determine if delta is significantly different from 0. In general, whenever there is a claim about difference, there should be a significance test (e.g., for Fig 3(b)). It appears that all the standard

deviations in the results are very high (Table 1, Table 2). Given the test set is so small, this is highly concerning.

<u>Reply</u>: We thank the reviewer for suggesting a statistical validation of the property changes. Following the recommendation, we performed two-sample t-tests for all scenarios and property control conditions to examine (i) whether the mean improvement is significantly above zero and (ii) whether it is statistically consistent with the requested numeric target. We added this result in Supplementary Information Table 4 as follows:

Supplementary Table 4: **Paired t-test results for property control scenarios.** For each scenario, both the maintained and target properties are listed with their mean Δ, standard deviation, t-statistics, and p-values. Two-tailed tests were applied to the maintained properties to verify that no significant deviation occurred, while one-tailed (directional) tests were used for the target properties to confirm whether their changes were statistically consistent with the intended direction of optimization.

Scenario	Condition	Maintain	Δ (mean ± SD)	t (vs 0)	p (vs 0)	Target	Δ (mean ± SD)	t (vs 0)	p (vs 0)	t (vs target)	p (vs target)
1	ΔMw 0 / ΔlogP +1	Mw	0.045 ± 6.53	0.22	0.83	logP	+0.831 ± 0.204	128.8	$< 10^{-60}$	-26.19	2.26×10^{-115}
1	ΔMw 0 / ΔlogP -1	Mw	-0.27 ± 6.19	-1.39	0.17	logP	-0.841 ± 0.217	-122.3	$< 10^{-60}$	+23.20	1.68×10^{-95}
2	ΔMw 0 / ΔQED +0.10	Mw	-0.13 ± 5.23	-0.78	0.44	QED	+0.067 ± 0.053	39.79	5.4×10^{-207}	-19.43	2.20×10^{-71}
2	ΔMw 0 / ΔQED +0.20	Mw	-0.58 ± 5.36	-3.37	7.8×10^{-4}	QED	+0.100 ± 0.062	49.98	7.6×10^{-272}	-50.34	9.29×10^{-274}
3	ΔQED 0 / ΔSA -0.50	QED	-0.026 ± 0.043	-17.01	1.4×10^{-55}	SA	-0.438 ± 0.361	-34.14	4.2×10^{-158}	+4.87	1.35×10^{-6}
3	ΔQED 0 / ΔSA -1.00	QED	-0.028 ± 0.045	-17.62	6.6×10^{-59}	SA	-0.719 ± 0.445	-45.42	5.3×10^{-223}	+17.77	1.01×10^{-59}

As summarized in Table S4, all cases showed highly significant p-values ($p \ll 0.001$) for improvement above zero, confirming that DeepBioisostere successfully modulates molecular properties in the desired direction. However, all tests for equivalence to the exact numeric targets (H_0: μ = target) were rejected, indicating that while the model achieved clear directional control, it tended to underachieve the numeric target values (approximately 50–88 % of the requested magnitude depending on the scenario).

This result is expected given that DeepBioisostere does not perform de novo molecule generation but rather modifies existing molecules under strong structural and bioactivity-preserving constraints. Achieving the exact target values becomes increasingly difficult when (a) the control involves multiple interdependent properties or (b) the molecular scaffold limits feasible changes in the target descriptor. Nevertheless, as shown across Scenarios 1-3, larger target requests resulted in proportionally greater shifts in the property distributions, confirming that the model captures the monotonic relationship between the control condition and the magnitude of achievable modification.

<u>Q4</u>. Regarding the comparison with Modof in Table 1: Modof is trained to optimize plogP, which is a combination of logP, molecule complexity and synthesizability (SAscore). While DeepBioisostere is trained to optimize logP, it is unfair to compare the plogP-optimized Modof with DeepBioisostere on logP. In addition, DeepBioisostere's training data is very different from the benchmark data for molecule optimization that Modof uses, and thus, there will be significant distribution shift issue for Modof. Given that DeepBioisostere's training data is much larger than Modof's, the authors need to train Modof using DeepBioisostere's training data and DeepBioisostere's objective, that is, to improve logP, not Modof's original objective to improve plogP, and then compare DeepBioisostere and Modof. The authors need to have fair comparisons among all the methods including SwissBioisostere and He et.al., making sure all the methods are optimized with respective to the same property and use same training data; it is unfair to compare methods that are not optimized for

DeepBioisostere's objective and claim they are worse than DeepBioisostere with respective to DeepBioisostere's objective.

<u>Reply</u>: We thank the reviewer for this valuable feedback regarding the fairness of the comparisons. The primary issue is that the deep learning-based methods included in our initial comparison (e.g., Modof) were not specifically designed for the task of bioisosteric replacement.

To address this issue, we have revised the benchmark design in a more fair and practical manner, as presented in Section 2.5. Recent *de novo* 3D molecular generative models can propose molecules with favorable binding affinity to a given target—referred to as "computational hit candidates" in the manuscript. However, a well-recognized limitation of these methods is that the generated molecules often suffer from poor drug-likeness and limited synthesizability, which hinders their utility in real drug discovery pipelines.

We therefore redesigned the benchmark to simulate this realistic setting: given computational hit candidates with promising binding affinity, the task is to improve their key drug-like properties while retaining their binding potential. In this revised benchmark, *DeepBioisostere* is compared against conventional database-mining strategies that search for analogues with similar binding affinity. The new benchmark study (Table 2) demonstrates that *DeepBioisostere* achieves superior performance in simultaneously controlling binding affinity and other critical molecular properties, highlighting its advantage over existing approaches in multi-property optimization.

Model	Strategy	Success Rate			
		QED	SAscore	Docking	Joint
DeepICL	Random	0.19	0.17	0.97	0.06
	Frequency-based	0.30	0.26	**0.99**	0.10
	MMPA-based	0.33	0.22	0.97	0.11
	DeepBioisostere	**0.69**	**0.80**	0.96	**0.56**
Pocket2Mol	Random	0.62	0.39	0.91	0.24
	Frequency-based	0.67	0.51	0.97	0.36
	MMPA-based	0.64	0.42	**0.98**	0.30
	DeepBioisostere	**0.75**	**0.74**	0.94	**0.56**
TargetDiff	Random	0.32	0.50	0.96	0.18
	Frequency-based	0.43	0.58	0.98	0.25
	MMPA-based	0.42	0.50	**0.99**	0.20
	DeepBioisostere	**0.83**	**0.87**	0.97	**0.74**
DecompDiff	Random	0.38	0.39	0.95	0.19
	Frequency-based	0.49	0.45	0.98	0.24
	MMPA-based	0.43	0.29	**0.99**	0.13
	DeepBioisostere	**0.79**	**0.87**	0.96	**0.68**

Table 2: **Success rate of in silico hit-to-lead workflow.** Starting from the model-derived hit candidates, four different replacement strategies are applied to achieve the goal of multi-property control—enhancing QED and SAscore while maintaining the docking score. The best success rate among strategies is highlighted in bold.

Q5. The pre-trained model of DeepBioisostere with all the model parameters and hyperparameters, as well as the processed training and test data, need to be published. The authors only published the code to process data and train models. Also, the new molecules with fragments replaced should also be published.

Reply: We thank the reviewer for highlighting this important point regarding reproducibility and statistical reliability. To ensure transparency and reproducibility, we have uploaded all necessary resources to our GitHub repository (https://github.com/Hwoo-Kim/DeepBioisostere/), including:

- the processed training and test datasets,
- the filtering procedure and resulting test molecule SMILES,
- the generated molecules with fragment replacements (SMILES), and
- the pre-trained model parameters and hyperparameters.

We have also verified that the repository runs correctly in independent computing environments. We hope this will allow the community to fully reproduce and further build upon our results.

Q6. There are quite a few multi-property optimization papers. In addition, though not specifically designed, Modof model can also be adapted to multi-property optimization tasks by altering its objective. However, this paper does not discuss them in the related work or compare with them as baselines. Below are examples for multi-property optimization papers:

a. Jin, Wengong, Regina Barzilay, and Tommi Jaakkola. "Multi-objective molecule generation using interpretable substructures." International conference on machine learning. PMLR, 2020.

b. Fu, Tianfan, et al. "Mimosa: Multi-constraint molecule sampling for molecule optimization." Proceedings of the AAAI Conference on Artificial Intelligence. Vol. 35. No. 1. 2021.

c. Loeffler, Hannes H., et al. "Reinvent 4: Modern AI–driven generative molecule design." Journal of Cheminformatics 16.1 (2024): 20.

More comparison results with multi-property optimization methods are needed.

Reply: We appreciate the reviewer's suggestion and agree that multi-property optimization is an active area of research. The cited works indeed address simultaneous optimization of multiple molecular properties. However, our study focuses specifically on bioisosteric replacement, where property control must be achieved while maintaining the molecule's bioactivity toward a target. The models mentioned, including Modof, are not designed to ensure bioactivity preservation or to operate within the structural constraints of bioisosteric substitution. Therefore, while we have added a discussion of these methods in the introduction for explanation, we did not include them as baselines, as their optimization objectives and problem formulations fundamentally differ from the scope of our work.

"Jin et al. [30], Fu el at. [31], and Loeffler et al. [32] employed standard optimization algorithms to refine molecular structures toward specific biological targets with low-cost labels, such as docking scores."

<u>Q7</u>. The authors claimed that DeepBioisostere is "a deep generative model for multi-property control with designing suitable bioisosteric replacements." However, DeepBioisostere essentially screens all the fragments in the pre-defined library in order to decide which fragment should be used as a replacement. This is not "generative" – it does not generate new fragments as Modof and many other generative models (e.g., JTNN) do; instead, it looks for and literally tries existing fragments. DeepBioisostere produces new molecules by replacing fragments; however, even in this sense, the notion of "generative" is much weaker than other molecule generation methods. Thus, "generative" is misleading and is a misconception to claim DeepBioisostere.

<u>Reply</u>: We thank the reviewer for raising this important point on the essence of generative modeling. *DeepBioisostere* calculates the likelihood of each candidate fragment in the fragment library given the starting molecule and the property control condition and generates new molecules by recombining these fragments. As such, our framework does not generate fragments *de novo* from scratch but rather generates molecules through the recombination of known fragments. We acknowledge that this may appear closer to a screening strategy than to unconstrained generation.

However, our model is designed to perform autonomous bioisosteric replacement, a strategy that is fundamentally defined by substituting one chemical moiety with another. While the fragments themselves are drawn from a pre-defined library, the final molecule (M') produced by this replacement is newly generated, which is not in any existing database. DeepBioisostere learns the conditional probability distribution $p(M'|M, C)$, which models the likelihood of a modified structure given the original molecule and the property-control objective. By sampling from this learned distribution, the model generates novel molecular structures—which did not exist previously—by attaching a context-appropriately selected fragment to the remaining molecular scaffold. We believe this process of learning a distribution and sampling from it to create new molecules aligns with the core concept of a generative model.

Finally, as discussed in SI Section 4.2 (*Statistics of the Fragment Library*), our fragment library comprises over 100,000 unique fragments, which provides sufficiently broad coverage of chemical space to support diverse generative outcomes beyond the chemical space of known molecules.

<u>Q8</u>. Section 2.4.2 appears very odd. First of all, the notations A->A and B->B are very confusing. Second, this exercise shows the importance of the chemical environment, which is general knowledge. It does not show whether DeepBioisostere can better leverage the environment, particularly automatically and in comparison with other methods. Also, figure

3(b) does not show a significant difference among all three cases in their delta logP; a significance test is needed.

Reply: We thank the reviewer for these valuable comments. First, we agree that the notations *A→A* and *B→B* were confusing. They are the cases where DeepBioisostere model is normally applied to original molecules, so they are not necessarily expressed with new notations such as A→A and B→B. In the revised manuscript, we simply denoted them as M_A' and M_B'. In the same way, A→B was changed to M_B'', and we revised its explanation to be clearer:

"Here, we forced our model to choose only the pyrazole groups as removal fragments to modulate a given target property, resulting in 100 unique modified structures. (M_A' and M_B' in Fig. 3(a)) For comparison, we retrieved the insertion fragments selected for molecule A and incorporated them into molecule B, resulting in another set of 100 unique molecules. (M_B'' in Fig. 3(a)) In the first two cases, M_A' and M_B', the individual chemical environments were directly engaged in the selection of insertion fragments. However, in the case of M_B'', the insertion fragments were selected based solely on the chemical environment of molecule A, without any involvement from molecule B."

Fig. 3 was revised accordingly:

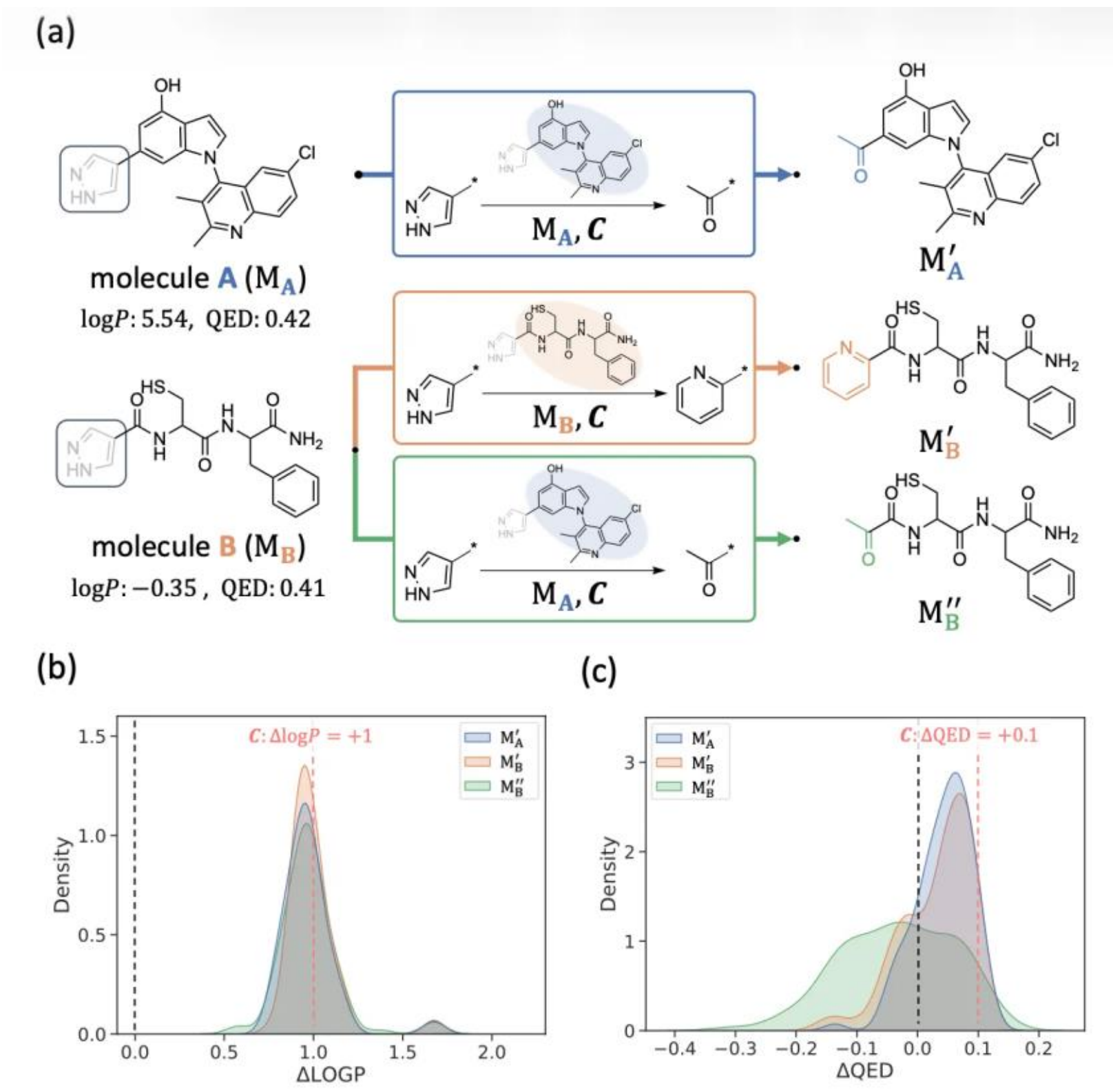

Fig. 3: **An example showing the significance of chemical environment during insertion fragment selection.** (a) Three replacement cases: within molecule A (M_A'), within molecule B (M_B'), and from molecule A to molecule B (M_B''). (b) $\Delta \log P$ distributions of modified molecules from each original molecule. (c) ΔQED distributions of modified molecules for each original molecule. In both (b) and (c), the black and red dashed lines denote the baseline implying original molecules' property values and property conditioning objectives, respectively.

To make this distinction clear, we deliberately examined two properties with different levels of chemical environmental dependence: logP (which is highly additive and thus weakly dependent on the global environment) and QED (which is non-linear and thus strongly dependent on the global environment). As the reviewer noted, Figure 3(b) shows that the differences in ΔlogP across the three cases are minor. This is the expected result and consistent with the additive nature of logP. An insertion fragment is expected to have a similar ΔlogP effect regardless of whether it is placed in molecule A or molecule B. By contrast, Figure 3(c) shows that QED exhibits strong dependence on the chemical environment. Inserting fragments selected to improve QED for molecule A into molecule B failed to improve QED and, in fact, worsened it. To emphasize that conventional approaches cannot capture such environment-dependent property control, we have revised the manuscript accordingly:

"*This example highlights the importance of considering the entire chemical environment, not just the removed fragment, when designing a suitable bioisosteric replacement for properties that depend on global molecular context. Such context-dependent property modulation cannot be handled by database-mining approaches, which rely solely on pre-defined fragment statistics. In contrast, DeepBioisostere explicitly learns to capture the non-linear relationships between fragments and their molecular environments, enabling context-aware bioisosteric replacements."*

Q9. In Discussion section, there are no discussions about the potential issues of DeepBioisostere.

Reply: We thank the reviewer for this valuable feedback regarding the lack of discussion on potential limitations. While *DeepBioisostere* offers the advantage of enabling multi-property control through a deep generative framework, it also has clear limitations. In particular, although our model can modulate molecular properties specified as control conditions, it inherits a general limitation of conditional generative models: conditioning is effective only for properties that can be reliably and consistently labeled. A promising direction to address this challenge is to leverage multi-task and transfer learning. By pretraining the model on properties with abundant labels (e.g., QED, logP, SA score) and transferring the learned molecular representations, the framework can better accommodate conditions with limited labels, such as specific ADMET properties. We have added this limitation to the revised Discussion section accordingly:

"While DeepBioisostere offers the advantage of enabling multi-property control --- including binding affinity --- through a deep generative framework, it also has still limitations. In particular, while our model can modulate molecular properties defined as control conditions, it shares a common limitation of conditional generative models: it can only effectively modulate properties with abundant and reliable labels. A promising direction to address this challenge is to leverage multi-task and transfer learning. By pretraining the model on properties with abundant labels (e.g., QED, logP, SA score) and transferring the learned molecular representations, the framework can better accommodate conditions with limited labels, such as specific ADMET properties. Such advancements could broaden the

applicability of DeepBioisostere to a wider range of drug discovery tasks and more challenging molecular design scenarios.”

Reviewer #1 (Remarks on code availability):

The pre-trained model of DeepBioisostere with all the model parameters and hyperparameters, as well as the processed training and test data, need to be published. The authors only published the code to process data and train models.

Reply: We appreciate the reviewer for emphasizing the importance of transparency and reproducibility in statistical evaluation. As mentioned in our response to Q5, we have uploaded and made publicly available all relevant resources in our GitHub repository (https://github.com/Hwoo-Kim/DeepBioisostere/), including the training data, test molecule filtering results and generated SMILES, as well as the trained model parameters. We have also verified that the model runs properly in different computing environments and provided detailed usage instructions in accompanying Jupyter notebook files for ease of use.

Reviewer 2.

Q1. I am curious about the Authors' definition of bioisosteres. I learned that bioisosteric replacements, as suggested by the name, are usually very subtle changes that do not change the shape or electronic properties of a compound so that it can preserve its bioactivity while optimizing other properties. The replacements proposed by DeepBioisostere are often more extensive and change the overall shape of the molecule. Would it not be considered scaffold hopping?

Reply: We thank the reviewer for highlighting this important point. We realized that in the previous version of the manuscript our definition of bioisosterism had been extended too broadly, beyond fragment pairs with similar bioactivity.

In our framework, the definition of bioisosteres fundamentally follows the database-mining approach, i.e., fragment pairs that have been experimentally shown in bioassays (e.g., ChEMBL) to exhibit similar levels of bioactivity. However, in the earlier version of the manuscript, we mistakenly included as “bioisosteres” all fragment pairs derived from matched molecular pairs (MMP) in ChEMBL that were structurally similar at the graph level, regardless of whether there was experimental evidence for comparable bioactivity. This overly broad definition neglected the critical role of experimental evidence and thus undermined the expectation that bioactivity would be preserved.

To address this concern, we reconstructed the training dataset. Specifically, we restricted the MMP exploration procedure to the pairs of molecules that were tested within the same bioassay and whose experimental bioactivities (represented by pChEMBL values) differed by no more than one order of magnitude (10-fold). As a result of this filtering, the number of extracted MMPs decreased from 24,163,198 to 2,874,580, successfully eliminating fragment pairs without evidence of bioactivity preservation and retaining only those with meaningful

experimental support. The manuscript has been revised accordingly to describe this data processing procedure and its outcome:

“To ensure that only MMPs preserving bioactivity were considered, pairs with |ΔpChEMBL| > 1 were filtered out. The final training dataset consists of a total of 2,874,580 MMPs specified about their corresponding bioisosteres.”

Q2. Following the logic in the previous point, demonstrating that DeepBioisostere can modify the molecular structure to optimize, e.g., solubility without changing the binding pose and predicted affinity would be an interesting case study that would prove the method useful in a more classical application of bioisosterism.

Reply: We thank the reviewer for this insightful suggestion. As noted in our response to Q1, your comments motivated us to strengthen the aspect of bioactivity preservation in our framework, and to further examine this point, we performed an additional analysis, now reported in Section 2.5 and Table 2.

To define a set of starting molecules with good binding affinity, we utilized several recently proposed state-of-the-art 3D molecular generative models—Pocket2Mol, DeepICL, TargetDiff, and DecompDiff. While these models are capable of producing molecules with favorable docking scores, they often fall short in terms of synthesizability and overall drug-likeness, limiting their applicability in practical drug discovery. We therefore refer to these molecules as *Computational Hit Candidates*. On this set, we applied *DeepBioisostere* to perform multi-property control tasks and conducted statistical evaluations.

Specifically, we targeted improvements in synthetic accessibility (SAscore) and drug-likeness (QED). To ensure that binding affinity was preserved during these modifications, we performed docking simulations on the modified molecules. Because such properties may exhibit trade-offs, it is essential to evaluate the extent to which multiple properties can be simultaneously improved. To this end, we defined a joint success metric, denoted as *Joint*, which measures the success rate of simultaneous improvement across all targeted properties.

Compared with conventional database-mining strategies, *DeepBioisostere* achieved similar or significantly greater improvements in individual properties across all the 3D generative models. Importantly, in terms of the *Joint* metric for multi-property optimization, our model demonstrated substantially higher success rates than the baseline methods. These results provide strong evidence that *DeepBioisostere* can not only perform desired property control but also remain consistent with the classical definition of bioisosterism, by exploring analogues that preserve bioactivity while optimizing other properties.

<u>Q3</u>. The motivation of the paper is clear. The automatic selection of the fragment for replacement is important for streamlining the lead optimization process. However, one sentence in the introduction is difficult to understand: "Also, the expectation of property changes based on the mined data may not work for more complicated properties that depend on complex relationships between internal moieties, such as synthesizability or drug-likeness." Could you please rephrase this sentence?

<u>Reply</u>: As the reviewer pointed out, the original sentence was too abstract and complex to understand. We have revised it as follows:

"Because such methods evaluate property changes based only on the exchanged fragments, they struggle to capture complex attributes such as synthesizability or drug-likeness, which inherently depend on the entire molecular context rather than local substitutions."

<u>Q4</u>. Most of the related work is described in the introduction, which puts this research in the context of other methods for molecular optimization and bioisosteric replacement. I recommend including a few articles about machine learning-based scaffold hopping, which present a fully automated procedure of molecular optimization. It would be interesting to see a few methods of this kind in the experiment presented in Table 1 as an example of different approach to molecular optimization. For example:

[1] Zheng, S., Lei, Z., Ai, H., Chen, H., Deng, D., & Yang, Y. (2021). Deep scaffold hopping with multimodal transformer neural networks. Journal of cheminformatics, 13, 1-15.

[2] Hu, C., Li, S., Yang, C., Chen, J., Xiong, Y., Fan, G., ... & Hong, L. (2023). ScaffoldGVAE: scaffold generation and hopping of drug molecules via a variational autoencoder based on multi-view graph neural networks. Journal of Cheminformatics, 15(1), 91.

<u>Reply</u>: We thank the reviewer for pointing out relevant works in the areas of molecular optimization and scaffold hopping. The suggested studies are indeed interesting contributions that apply deep generative models to scaffold hopping, enabling the discovery of novel chemical structures with similar bioactivity. They share similarities with our work in that they address the broader problem of molecular optimization by searching for molecules with preserved biochemical properties.

However, the primary objective of *DeepBioisostere* differs substantially from scaffold hopping. While scaffold hopping methods seek to generate structurally distinct molecules that maintain similar bioactivity, *DeepBioisostere* instead focuses on making minimal structural changes—replacing only a single fragment with a bioisostere—in order to preserve favorable properties of the original molecule while simultaneously optimizing additional properties, including binding affinity.

Because of this fundamental difference in objectives, a direct experimental comparison would not be fair. Nevertheless, given their conceptual relevance in exploring structurally related analogues, we have included these methods in the introduction rather than as baselines.

“Zheng et al. [39] and Hu et al. [40] demonstrated that deep learning methods can be effectively applied to scaffold hopping, generating alternative scaffolds while maintaining biological activity.”

<u>Q5</u>. While the approach described in the manuscript appears to be novel, I do not think the following sentence is true: “Note that DeepBioisostere is the first generative approach to the bioisosteric replacement for molecular optimization.” There are already at least a few examples of other generative models that can perform bioisosteric replacement, e.g., Shan and Ji [3] generate SMIRKS rules with recurrent neural networks, and Maziarka et al. [4] formulate bioisosteric replacement as a domain adaptation task. These approaches are significantly different, but nonetheless, they are examples of generative models for bioisosteric replacement.

[3] Shan, J., & Ji, C. (2020). MolOpt: a web server for drug design using bioisosteric transformation. Current computer-aided drug design, 16(4), 460-466.

[4] Maziarka, Ł., Pocha, A., Kaczmarczyk, J., Rataj, K., Danel, T., & Warchoł, M. (2020). Mol-CycleGAN: a generative model for molecular optimization. Journal of Cheminformatics, 12(1), 2.

<u>Reply</u>: We thank the reviewer for this helpful feedback on clarifying the originality of our work. We carefully reviewed the two suggested papers and acknowledge that they indeed present generative models that perform bioisosteric replacement.

Reference [3] shares a similar goal with Ertl’s work [REF28 in the revised manuscript], which we had cited in the introduction: it generates bioisosteric replacements for given molecular fragments using a language model, where the bioisosteres used for training are obtained through database mining. Reference [4] demonstrates a generative model that performs a specific bioisosteric replacement (e.g., converting a CN moiety into a CF_3 moiety) formulated as a domain adaptation task. Both are clear examples of generative models for bioisosteric replacement.

Still, these approaches differ from *DeepBioisostere* in important ways. In particular, the model in [3] does not explicitly consider the chemical environment surrounding a bioisosteric replacement, making it less suitable for complex multi-property control. Meanwhile, the work in [4] focuses on a single bioisosteric transformation and does not ensure structural similarity to the original molecule, which may struggle with the preservation of crucial properties already present in the original compound.

In light of this, we agree with the reviewer that our original statement—"*DeepBioisostere* is the first generative approach to bioisosteric replacement for molecular optimization"—was overstated. We have revised the introduction to adopt a more adequate description:

"Here, we propose DeepBioisostere, a deep generative model for autonomous bioisosteric replacement with fine control over multiple molecular properties in an end-to-end manner, selecting modification sites and generating context-aware substitution."

And we have added them to the introduction as related works to identify potential bioisosteric replacements, rather than including them as baselines:

"For example, Etrl [28], and Shan and Ji [29] applied neural networks to fragment-level data derived from ChEMBL, demonstrating the ability to identify potential bioisosteric replacements beyond the training set."

[REF28 in the revised manuscript] Ertl, P.: Identification of bioisosteric substituents by a deep neural network. Journal of Chemical Information and Modeling 60(7), 3369–3375 (2020) https: //doi.org/10.1021/acs.jcim.0c00290

Q6. The results described in Sections 2.2 and 2.3 are impressive and compelling. However, only very simple properties are considered in these experiments. I know these are standard descriptors often used in the literature to evaluate machine learning models, but I wonder how easy it is to apply more advanced molecular properties here. If I understand correctly, these properties need to be known for all training molecules because they are a part of the conditional distribution p(M'|M,C). It is only possible because cLogP, QED, and SA can be computed in milliseconds. The task becomes much more difficult if someone wants to use wet-lab bioactivity data (virtually impossible to achieve for almost a million ChEMBL molecules) or even more advanced simulated data like molecular docking results. Applying machine learning models could be a solution, but training an ML model with an applicability domain as big as the whole ChEMBL dataset is still infeasible. If my understanding is correct, this needs to be described as a limitation of the proposed model. Could you suggest any solution to that problem? For example, can you train DeepBioisostere with partially labeled data?

Reply: We thank the reviewer for this thoughtful comment. The reliance on large-scale labeled data is indeed one of the key limitations of *DeepBioisostere* as a conditional generative model, and more broadly of supervised learning approaches in general. While simple descriptors such as cLogP, QED, and SA can be computed efficiently for millions of molecules, it is far more challenging to scale to experimentally measured bioactivity data or computationally intensive properties such as docking scores.

To address this challenge, two promising directions can be considered:

1. **Semi-supervised learning.** Several studies have demonstrated that semi-supervised learning schemes can be successfully applied to conditional molecular

generation [REF1, REF2]. By leveraging large amounts of unlabeled molecules alongside limited labeled data, such approaches can mitigate the dependency on fully annotated datasets.

2. **Transfer learning.** Another viable strategy is to pretrain on properties with abundant labels (e.g., QED, logP, SA score) and then transfer the learned molecular representations to tasks involving more complex or sparsely labeled properties, such as bioactivity or ADMET endpoints. This multi-task or transfer learning approach has been shown to improve the generalization of molecular generative models when labeled data are scarce. [REF3, REF4]

While both strategies may not fully match the performance achievable with large-scale labeled datasets, they represent practical ways to extend *DeepBioisostere* to more challenging property-control tasks. We have added this limitation and the corresponding potential solutions to the Discussion section of the revised manuscript:

"While DeepBioisostere already demonstrates the ability to control multiple molecular properties through a deep generative framework, its design also opens promising directions for further enhancement. In particular, extending the model with multi-task and transfer learning could enable it to handle conditions with limited or noisy labels more effectively. Pretraining on widely available or easy-to-label molecular properties, such as QED, logP, and SA score, and transferring the learned representations to tasks involving complex endpoints, like ADMET properties, would enable the framework to generalize across a broader chemical and biological space. Such advancements could further strengthen the model's applicability to diverse drug discovery pipelines, facilitating more accurate and data-efficient molecular design in increasingly realistic and challenging scenarios."

[REF1] Xie et al., "CoG: Conditional molecular generation with contrastive semi-supervised learning," NeurIPS 2021

[REF2] Kang, Seokho, and Kyunghyun Cho. "Conditional molecular design with deep generative models." *Journal of chemical information and modeling* 59.1 (2018): 43-52.

[REF3] Wang, Xun, et al. "PETrans: De novo drug design with protein-specific encoding based on transfer learning." *International Journal of Molecular Sciences* 24.2 (2023): 1146.

[REF4] Yamaguchi, Shin'ya, et al. "Transfer learning wih pre-trained conditional generative models." *Machine Learning* 114.4 (2025): 96.

Q7. All the figures in the manuscript are very informative, especially Figures 2 and 3, which provide readers with a better understanding of the inner workings of the proposed algorithm. I also enjoyed the case study on targeting the SARS-CoV-2 main protease mutant. This well-designed experiment showed an example of a real-life application even though only simple computed properties like cLogP could be optimized.

<u>Reply</u>: We thank the reviewer for the positive feedback on our work. Figures 2 and 3 were designed to illustrate and analyze the internal mechanism of *DeepBioisostere*, showing how the model operates and which factors it considers when performing fragment replacements. We believe that such analyses of model behavior and performance from multiple perspectives contribute to the development of AI models that are easier to understand and more trustworthy.

The final case study was originally intended to demonstrate that *DeepBioisostere* can be applied to practical problem settings through appropriate use of property control conditions. However, as the reviewer noted in Q1 and Q2, a stricter adherence to the definition of bioisosterism requires ensuring bioactivity preservation. To address this, we reprocessed the data and revised the manuscript accordingly, which necessitated more rigorous statistical analyses of bioisosteric generation. As a result, Section 2.5 was modified from the previous SARS-CoV-2 main protease mutant case study to the current statistical analysis of multi-property control on computational hit candidates.

Nevertheless, *DeepBioisostere* remains capable of proposing molecules with improved drug-likeness or bioactivity when appropriate property control conditions are specified, making it a flexible tool for diverse practical applications.

<u>Q8</u>. The architecture design of DeepBioisostere is well thought-out. I am curious about a few design decisions that seem nonstandard in the machine learning practice. The Authors can comment on these aspects.

a) Why is the a_i term inside the sum in Equation 2? This term is independent of a_j, so it can be moved outside the sum. Now, the signal from the central node is stronger, but it can be canceled by the trainable weights. However, it seems inefficient from the computation perspective.

b) Why is GRU used in Equation 3? Recurrent neural networks are typically used to process sequences. What is the intuition behind using GRU instead of a simple activation function?

c) Equation 5 treats the atoms inside the replaced fragment the same way as the atoms in the rest of the molecule (there is no direction of the bonds). Could adding information about the direction (inside vs outside the replaced fragment) help the model learn better representation?

d) A binary cross-entropy is used in the loss functon(Eq. 12). Why have you decided not to use a multi-class cross-entropy? What if two MMPs are found for a molecule, and two different fragments are positive samples?

e) A negative sign is typically used on the right-hand side of equations when defining loss functions (Eqs. 12-14).

Reply: We sincerely thank the reviewer for the deep and thoughtful feedback on the design of our model architecture. We carefully considered the points raised and addressed them in detail below.

a & b) *DeepBioisostere* is fundamentally based on the Message Passing Neural Network (MPNN) architecture, which has demonstrated strong performance across diverse problems in chemical domains [REF5]. Specifically, we follow Eqs. (1) and (2) of the original MPNN paper, reproduced below for reference:

$$m_v^{t+1} = \sum_{w \in N(v)} M_t(h_v^t, h_w^t, e_{vw}) \qquad (1)$$

$$h_v^{t+1} = U_t(h_v^t, m_v^{t+1}) \qquad (2)$$

, where h and e are node and edge embedding vectors, M_t is the t-th message passing function, and U_t is the t-th node update function. Prompted by the reviewer's comment, we examined a wide range of MPNN variants and found that most commonly, the message function M_t depends on h_w^t and e_{vw} (or both), rather than on the embedding of the receiving node h_v^t. Following this suggestion, we trained an alternative message passing architecture in which the receiver features (a_i^t and f_n^t in our manuscript's notation) are excluded from Eqs. (2) and (6). This modification consistently led to a significantly lower validation loss across the three property-control tasks reported in Table 1. The final validation losses of models trained with the original and revised message passing functions are summarized below:

	Previous message passing	New message passing
1. MW & LOGP	0.768	**0.741**
2. MW & QED	0.843	**0.826**
3. QED & SAscore	0.929	**0.864**

Accordingly, we revised the architecture to adopt the reviewer's recommended message passing formulation, and Eqs. (2) and (6) in the manuscript have been updated as follows:

$$\mathbf{m}_i^t = \sum_{a_j \in \mathcal{N}(a_i)} W_1^a \mathbf{a}_j^t + W_2^a \mathbf{b}_{ij}, \qquad (2)$$

$$\mathbf{m}_n^t = \sum_{\hat{f}_m \in \mathcal{N}(\hat{f}_n)} W_1^f \mathbf{f}_m^t + W_2^f \mathbf{e}_{nm}, \qquad (6)$$

Regarding the update function, we employed the GRU following the design of the Gated Graph Neural Network (GG-NN) [REF6]. Although GRUs are more widely known in the context of recurrent neural networks, their essential role—balancing the influence of long-term memory with new information—closely parallels the purpose of the update function in MPNNs, namely determining how to combine the current hidden state h^t with the new incoming message m^{t+1}. Moreover, because GRUs inherently include nonlinear transformations, they can also serve as effective activation functions. For these reasons, we selected GRUs as the update mechanism in *DeepBioisostere*.

[REF5] Gilmer, Justin, et al. "Neural message passing for quantum chemistry." *International conference on machine learning*. Pmlr, 2017.

[REF6] Li, Yujia, et al. "Gated graph sequence neural networks." *arXiv preprint arXiv:1511.05493* (2015).

c) We sincerely thank the reviewer for this valuable idea for obtaining richer representations. We understand the key suggestion as leveraging directional edges in the message passing process so that information about the fragment selected for removal can be better incorporated. Unfortunately, in our current modeling framework, the representation construction process concludes by Eq. (8), and the decisions for removal fragment, insertion fragment, and attachment orientation are subsequently made based on this representation. Since Eq. (5) precedes the removal fragment selection step, it is not possible to directly incorporate removal fragment information at that stage.

That said, as shown in Eq. (10), the embedding vector of the fragment selected for removal is explicitly used, ensuring that both the fragment representation and the information about its selection are integrated into the prediction and generation processes.

d) We thank the reviewer for this thoughtful question, which helped us clarify the details of our training loss. As the reviewer noted, the target distribution in our setting is a multinomial distribution, which can be naturally viewed as a multi-label classification problem. In such cases, it is indeed common to employ a multi-class cross-entropy loss.

In our framework, however, we adopted a binary cross-entropy (BCE) loss with negative sampling, a technique widely used in language models such as *word2vec* [REF7]. This strategy is particularly effective when the number of classes is very large and has also been successfully applied in fragment-based molecular generation models [REF8].

There are two main reasons for this choice:

1. The fragment library contains hundreds of thousands of classes, each represented not by a simple index but by a fragment embedding (as in Eq. (10)). In this case,

computing a full multi-class cross-entropy loss over the entire library would be highly inefficient.

2. In situations where multiple positive labels exist, say fragments A and B, alternating between training with A as positive and B as negative, and vice versa, naturally drives the model to converge toward assigning equal probability (≈0.5) to both. As shown in Eqs. (12)–(14), each corresponds to the loss of a single data point, while the overall training loss is defined as their expectation. This formulation resolves the concern raised by the reviewer.

To make this point explicit, we have added more explanations below the Eq. (13) as follows:

"One potential concern is that, for a given molecular structure and its removal fragment pair, if there are two distinct ground-truth insertion fragments, one could mistakenly be sampled as a negative fragment during training. However, since the final training objective is defined as the expectation over the entire dataset, the model will ultimately assign equal probabilities to both fragments."

[REF7] Church, Kenneth Ward. "Word2Vec." *Natural Language Engineering* 23.1 (2017): 155-162.

[REF8] Seo, Seonghwan, Jaechang Lim, and Woo Youn Kim. "Molecular generative model via retrosynthetically prepared chemical building block assembly." *Advanced Science* 10.8 (2023): 2206674.

<u>e)</u> We thank the reviewer for pointing out the unconventional aspect of our loss formulation. As suggested, we have revised Eqs. (12)–(14) accordingly.

<u>Q9</u>. I recommend including an ablation study in the manuscript that shows the proposed model's results when other architectures or sampling strategies (Section 4.4) are used. This experiment would explain the design decisions described above and provide a better understanding of the model.

<u>Reply</u>: We thank the reviewer for this helpful suggestion regarding model evaluation. As the reviewer points out, analyzing how the model's predictions change when alternative architectures or sampling strategies are used provides valuable insight into the underlying design choices.

To address this, we conducted an ablation study focusing on the model architecture. The strong performance of *DeepBioisostere* in property control via bioisosteric replacement stems from its ability to capture fragment-level context. Because all decisions in *DeepBioisostere* are made at the fragment level, we introduced the subgraph AMPN—a fragment embedder with shared weights across the given molecule and the fragment library.

This component was designed to enhance the understanding of relationships between fragments and properties within the molecular context.

We added an ablation study section to the Supplementary Information (Section 5.1) SI Figure 4 to examine the role of the subgraph AMPN. As shown in the ablation study, removing the subgraph AMPN led to a clear drop in performance for all multi-property control scenarios, confirming that the shared fragment embedder plays a significant role in connecting removal and insertion fragment selection with property control.

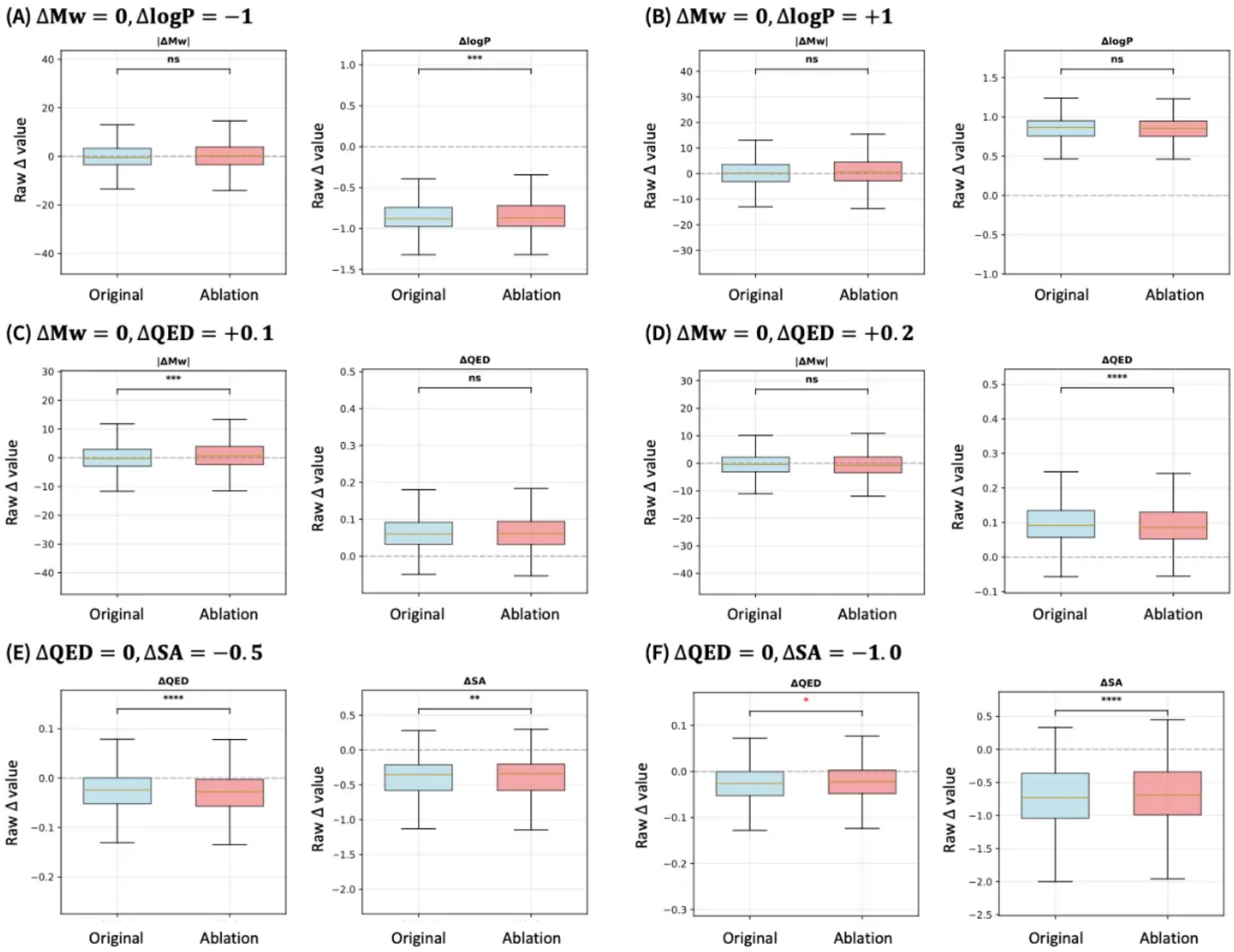

Supplementary Figure 4: Paired t-test results to compare multi-property control performances; ns $P > 0.1$, *$P < 0.1$, **$P < 0.05$, ***$P < 0.01$, and ****$P < 0.001$. Black stars designate the original model statistically achieves higher performance, and red ones mean the ablated model exhibits better results. Used input property control conditions are shown in sub-titles. (A,B) Scenario where MW and $\log P$ are modulated simultaneously. (C,D) Scenario where MW and QED are modulated simultaneously. (E,F) Scenario where QED and SA are modulated simultaneously.

Reviewer #2 (Remarks on code availability):

I managed to install the conda environment and run the code in the example notebook. Unfortunately, a trained model is not provided, and the data used for training is not available yet, so I could not test the generator. The usage is properly described in the README file, which includes model training and inference, but jupyter notebook needs to be downloaded to run the example in the ipynb file (this dependency is not included in the README file nor in the dependencies script). The code is organized in a clear structure. The code that reproduces plots in Figures 2, 3, and 4 is provided. I have not tried training the model from scratch because the training data is not provided, which makes the results in the paper irreproducible.

<u>Reply</u>: We appreciate the reviewer for emphasizing the importance of transparency and reproducibility in statistical evaluation. We have uploaded and made publicly available all relevant resources in our GitHub repository (https://github.com/Hwoo-Kim/DeepBioisostere/), including the training data, test molecule filtering results and generated SMILES, as well as the trained model parameters. We have also verified that the model runs properly in different computing environments and provided detailed usage instructions in accompanying Jupyter notebook files for ease of use.

<u>Reviewer 3.</u>

<u>Comment</u>. The authors present a deep generative model that automatically replaces fragments in small molecules with the purpose of optimizing calculated properties, while keeping others unchanged, relevant for drug discovery. The authors stipulate that their method is generating bioisosteric replacements that go beyond what is known from bioisostere databases or the medicinal chemistry literature at large.

In the scientific literature and by practitioners at large, the term bioisosteric replacement is used to describe the replacement of a part of a bioactive molecule with another substructure of similar size and similar properties. The term is generally applied to replacements that have been observed experimentally exhibiting an effect of maintaining experimental properties consistently across multiple examples. For instance, **Ertl requires at least five or more experimental examples used with limited spread in observed property differences (<1 log unit) before he is comfortable calling a replacement a bioisostere** [ref 29]. The authors attempt to extend the concept of bioisosteres beyond experimentally observed property similarity data to calculated property data. As a result, the number of potential ‘bioisosteric’ replacements goes up significantly. **In doing so, the authors leave the idea of experimental validation behind, extending the concept of bioisosteres instead to all matched molecular pairs (MMPs) of compounds with bioactivity <=10uM, regardless of experimentally validated property maintenance IN THE SAME ASSAY.** In my opinion, this extension to calculated-only properties, without consideration of experimental data, is not useful as it softens the term bioisostere at best and almost completely deprives it of its meaning at worst. Rather, it extends the term bioisostere to that

of MMPs (of compounds with any bioactivity against in at least one assay), which are not the same.

If I take the term ‘bioisostere’ and its underlying notion out of consideration for a moment, I see an algorithm and method that is not unlike other generative AI reinforcement models, such as REINVENT4, that generate compound ideas driven by a fitness function that can easily incorporate the parameters chosen by the authors of the manuscript, as they relate to logP, QED, MW, synthesizability score, etc. For this purpose, I do not see a particular advantage of limiting the generative AI to matched molecular pairs derived from Chembl. Therefore, I do not see a meaningful novelty demonstrated in the approach presented in the manuscript.

The author’s claim of their method outperforming data mining approaches for bioisosteres is not supported by the data as the author’s approach is not strictly searching for established bioisosteres but for hypothetical replacements that are not proven to be bioisosteres yet.

While the authors show that their method generated compounds with the desired calculated properties, **the comparison to other approaches such as Swissbioisostere is not done at equal footing as Swissbioisostere includes statistical information on the impact of replacements on bioactivities, while the author’s method does not seem to take such data into account**.

The authors apply their method to identifying inhibitors of the 3CLpro E166V mutant. None of the suggested inhibitors have been synthesized and tested. **The claims of improved properties of the putative inhibitors are not supported by experimental evidence**.

For the above reasons, I believe that the manuscript is not suitable for publication in Nature Communications.

Reply: We sincerely thank the reviewer for these constructive and thought-provoking comments regarding the definition and statistical foundation of our work. Their feedback made us recognize that in the previous version of the manuscript, our definition of bioisosterism had been extended too broadly, beyond fragment pairs with experimental evidence of bioactivity preservation. By definition, bioisosteres should be grounded in experimentally validated fragment pairs that exhibit similar bioactivity. This principle, as emphasized in Ertl’s work and other database-mining approaches, is fundamental.

In response, we have reconstructed our training dataset to ensure stricter adherence to this definition. Specifically, we restricted matched molecular pair (MMP) analysis to pairs of molecules from the ChEMBL database that were tested in the same assay and exhibited experimental bioactivities (pChEMBL values) within one log unit of each other. Through this restriction, the number of **training pairs was reduced from 24,163,198 to 2,874,580,** thereby successfully **eliminating approximately 85% of molecular pairs without experimental support** and retaining only those with stronger evidence of preserving bioactivity.

During the reconstruction of the training dataset, determining how many occurrences were required to consider a fragment pair as bioisosteric was a critical decision. As the reviewer noted, this threshold directly affects the statistical reliability of the dataset and, consequently, of the model trained on it. However, from a generative modeling perspective, increasing the threshold substantially reduces the number of available training pairs, thereby shrinking chemotype coverage and limiting the diversity of property contexts accessible to the model. In our preprocessing pipeline, increasing the occurrence threshold from one to five reduces the number of fragment pairs from 2,874,580 to 1,185,067. Because our objective is to learn transferable edit patterns under multi-property conditioning, maintaining a sufficient scale and diversity of training examples is essential for generalization in designing and identifying bioisosteric replacements. We therefore adopted the one-time occurrence criterion as the default for model training, which strikes a practical balance between data quantity and quality without overextending the claim of experimentally validated bioisosteres. Furthermore, our training objective inherently assigns frequency-dependent weights; rarely observed fragment pairs are still given proportionally lower preference during learning.

With this revised dataset grounded in experimental evidence, *DeepBioisostere* goes beyond simply controlling calculated properties such as logP, MW, QED, or SA score. It is also able to preserve the bioactivity of the given molecule without requiring any additional task-specific training for a new biological target. To our knowledge, no previous molecular optimization method has demonstrated such end-to-end capability regarding binding affinity. Reinforcement-learning-based frameworks such as REINVENT4[REF9] and generative optimization models such as JTVAE[REF10] and Modof [REF11] **all require additional target-specific training in order to account for bioactivity**. In contrast, ***DeepBioisostere*** **can directly explore bioactivity-preserving replacements while simultaneously optimizing other properties without target-specific labels, which represents a unique contribution.** We greatly appreciate the reviewer's feedback, which has strengthened both the reliability of our training data and the demonstrated capability of the model to control bioactivity.

Finally, we acknowledge the reviewer's concern regarding the previous case study on the SARS-CoV-2 3CLpro E166V mutant. We agree that it is insufficient to claim improvements in binding affinity without experimental validation, and that such an example risk diverting attention away from the core contribution of our model. While *DeepBioisostere* could be applied to specific targets by manually selecting the replacement site, as in the original case study, its primary novelty lies in its ability to perform end-to-end multi-property control in a target property-agnostic manner. For this reason, we replaced the SARS-CoV-2 case study with a new task (Section 2.5, Table 2) that **statistically evaluates multi-property control—including binding affinity—on diverse *computational hit candidates*.** In this revised analysis, *DeepBioisostere* achieved a much higher success rate than conventional bioisosteric approaches. We believe this demonstrates that our model, unlike conventional methods, can capture the global molecular context and design bioisosteric replacements that satisfy property control conditions.

[REF9] Loeffler, Hannes H., et al. "Reinvent 4: Modern AI–driven generative molecule design." Journal of Cheminformatics 16.1 (2024): 20.

[REF10] Jin, Wengong, Regina Barzilay, and Tommi Jaakkola. "Junction tree variational autoencoder for molecular graph generation." *International conference on machine learning*. PMLR, 2018.

[REF11] Chen, Ziqi, et al. "A deep generative model for molecule optimization via one fragment modification." *Nature machine intelligence* 3.12 (2021): 1040-1049.

Dear editor,

Please find the enclosed manuscript for your consideration to publish in *Nature Communications* as an article entitled "**Autonomous Bioisosteric Replacement for Multi-Property Optimization in Drug Design**", authored by Hyeongwoo Kim, Seokhyun Moon, Wonho Zhung, Shinwoo Kim, Jaechang Lim, and Woo Youn Kim.

We sincerely appreciate you and the reviewers for their thoughtful and constructive feedback on our revised manuscript (Manuscript ID: **NCOMMS-24-20570B**). We are grateful that the reviewers found our previous revisions to have improved the manuscript, and we have carefully addressed the remaining points raised by the editor and reviewers in this additional revision.

In particular, we have substantially clarified the statistical analysis of the ablation study. Following the editor's and reviewer's comments, we added more detailed summary statistics to help readers better understand the magnitude and consistency of the observed differences. We also revised the relevant text to avoid overstating the statistical significance of the ablation results and to clarify the interpretation of these statistical analyses. These changes are now reflected in Supplementary Information Section 5.1 and the newly added Supplementary Table 3, which provide additional statistical context for Supplementary Figure 4.

We have also addressed the remaining minor issues raised by Reviewer #2, including clarification of the notation and correction of the typographical error in the Supplementary Information. In addition, we have prepared an updated Source Data file to support the numerical values underlying the relevant display items, including the newly added statistical summaries.

We believe that these revisions have addressed all remaining scientific and editorial concerns raised during this round of review and have further strengthened the transparency, statistical rigor, and reproducibility of the manuscript.

We appreciate your continued consideration of our revised manuscript and look forward to hearing from you.

Sincerely yours,

Woo Youn Kim,

Professor of Chemistry, KAIST

Point-by-point replies to the feedback are as follows.

Editor's comment.

Q1. You will see that, while the reviewers find that your revisions improved the manuscript, some important points remain to be addressed. In particular, we agree with Reviewer #1 in that more details on the statistics should be included to confirm the significance of the ablations - we note this is particularly important since P-values are only significative in 6/12 tasks, despite your text saying the additions are "highly significative in all scenarios". Please revise your manuscript, addressing all the remaining issues raised by the reviewers.

Reply: We sincerely thank the editor for this important comment. We agree that the statistical analysis of the ablation study required a more careful and quantitative description, and that our previous wording overstated the statistical significance of the ablation results.

To address this point, we revised the relevant text in the Supplementary Information to avoid claiming that the ablation results are "highly significant in all scenarios." Instead, we now provide a more balanced interpretation of the statistical results, distinguishing the magnitude and consistency of the observed differences from their statistical significance. In addition, following the reviewer's suggestion, we added a new summary table for the ablation study as Supplementary Table 3. This table reports the paired values used for the t-test analysis in each task, along with the corresponding p-values, to show the consistency of the property differences between the original and ablated models.

Accordingly, we revised our interpretation of the ablation results to better reflect the original purpose of the experiment. Rather than treating the results as 12 independent single-property tests, we now interpret them in the context of six multi-property control tasks, where two coupled molecular properties must be considered together. From this perspective, the original DeepBioisostere model generally outperforms or matches the ablated model, suggesting that the original architecture provides more favorable trade-offs for jointly controlled molecular properties.

These revisions are reflected in Supplementary Information Section 5.1 and the newly added Supplementary Table 3, which together provide additional context for interpreting Supplementary Figure 4. We believe that these additions clarify both the statistical significance and the revised interpretation of the ablation results, while avoiding an overstatement of the model's advantage.

Task	n	Property	Objective	Original	Ablation	Paired difference	P value	Preferred
A	995	MW	minimize $\|\Delta\text{MW}\|$	4.5212	4.6471	-0.1259 ± 3.5714	0.133	ns
		log$P$	maximize $-\Delta\log P$	0.8419	0.8285	0.0134 ± 0.1503	0.00245	Original
B	995	MW	minimize $\|\Delta\text{MW}\|$	4.6666	4.7833	-0.1166 ± 3.9336	0.175	ns
		log$P$	maximize $\Delta\log P$	0.8330	0.8325	0.0005 ± 0.1332	0.453	ns
C	979	MW	minimize $\|\Delta\text{MW}\|$	3.7701	4.0906	-0.3205 ± 3.5469	0.00240	Original
		QED	maximize ΔQED	0.0682	0.0689	-0.0006 ± 0.0302	0.254	ns
D	979	MW	minimize $\|\Delta\text{MW}\|$	3.8040	3.9290	-0.1251 ± 3.6787	0.144	ns
		QED	maximize ΔQED	0.1001	0.0945	0.0056 ± 0.0313	1.86×10^{-8}	Original
E	793	QED	minimize QED drop	0.0328	0.0361	-0.0033 ± 0.0242	6.23×10^{-5}	Original
		SA	maximize $-\Delta$SA	0.4421	0.4314	0.0107 ± 0.1523	0.0244	Original
F	793	QED	minimize QED drop	0.0345	0.0329	0.0015 ± 0.0313	<u>0.0823</u>	<u>Ablation</u>
		SA	maximize $-\Delta$SA	0.7233	0.6903	0.0330 ± 0.1598	4.42×10^{-9}	Original

Supplementary Table 3: Summary statistics for the ablation study in Supplementary Figure 4. Original and ablation columns report objective-aligned Δ values averaged over paired input molecules. Paired differences are computed as original minus ablation (mean $\pm$ SD). For minimization objectives, negative paired differences favor the original model; for maximization objectives, positive paired differences favor the original model. P-values are obtained from one-sided paired t-tests. By default, the alternative hypothesis tests whether the original model outperforms the ablation. For underlined entries, the direction of the alternative hypothesis is reversed to test whether the ablation outperforms the original; therefore, smaller P-values in these cases indicate stronger evidence in favor of the ablation model. The "Preferred" column reports the model favored by the statistical test, with significance thresholds defined as ns $P > 0.1$, $^*P < 0.1$, $^{**}P < 0.05$, $^{***}P < 0.01$, and $^{****}P < 0.001$.

(SI page 10) *"The results in Supplementary Fig. 4 and Supplementary Table 3 show that the DeepBioisostere model generally outperforms or matches the ablated model across the six multi-property control tasks. While statistically significant differences (P < 0.05) are observed in 6 out of the 12 individual hypothesis tests, these results should be interpreted in the context of paired multi-property optimization, where each task simultaneously evaluates two coupled molecular properties with inherent trade-offs. In most tasks, the original model achieves either statistically significant improvement in one property while maintaining comparable performance in the other, indicating that the original architecture preserves more favorable trade-offs between jointly controlled molecular properties."*

<u>Reviewer #2.</u>

<u>Q1</u>. Supplementary Figure 4 does not clearly show the difference between the two distributions. The boxplots look very similar even when P<0.001 (because the paired t-test is used, even small but consistent differences between paired values may be statistically significant). Reporting, for instance, the mean difference in delta values between the original model and its ablation, along with the standard deviation, would help readers understand why the original model is significantly better than the ablated one.

<u>Reply</u>: We thank the reviewer for this helpful suggestion. We agree that Supplementary Figure 4 alone does not fully convey the paired nature of the statistical comparison, especially because visually similar distributions can still yield statistically significant results when the paired differences are small but consistent.

To make the ablation results easier to interpret, we added a new Supplementary Table 3 summarizing the paired comparison between the original DeepBioisostere model and the

ablated model. For each task and evaluated property, the table reports the mean objective-aligned property shift for both models, the mean paired difference between the original and ablated models, the standard deviation of the paired differences, and the corresponding p-values. These additional statistics clarify the magnitude and consistency of the paired differences underlying Supplementary Figure 4.

We also revised the accompanying text in Supplementary Information Section 5.1 to interpret the results in the context of six multi-property control tasks, rather than as 12 independent single-property tests. This revision better reflects the original purpose of the ablation experiment, where two coupled molecular properties must be considered together, and clarifies that the original architecture generally provides more favorable trade-offs for jointly controlled molecular properties.

(SI page 10) *"Although the boxplot distributions in Supplementary Figure 4 appear visually similar, the paired differences reveal small but consistent shifts across molecules (e.g., ΔlogP: +0.0134 ± 0.1503 in Task A), which lead to statistically significant results under the paired t-test. These results highlight that the significance arises from consistency across paired samples rather than large absolute differences."*

Task	n	Property	Objective	Original	Ablation	Paired difference	P value	Preferred
A	995	MW	minimize $\lvert\Delta\text{MW}\rvert$	4.5212	4.6471	-0.1259 ± 3.5714	0.133	ns
		log$P$	maximize $-\Delta\log P$	0.8419	0.8285	0.0134 ± 0.1503	0.00245	Original
B	995	MW	minimize $\lvert\Delta\text{MW}\rvert$	4.6666	4.7833	-0.1166 ± 3.9336	0.175	ns
		log$P$	maximize $\Delta\log P$	0.8330	0.8325	0.0005 ± 0.1332	0.453	ns
C	979	MW	minimize $\lvert\Delta\text{MW}\rvert$	3.7701	4.0906	-0.3205 ± 3.5469	0.00240	Original
		QED	maximize ΔQED	0.0682	0.0689	-0.0006 ± 0.0302	0.254	ns
D	979	MW	minimize $\lvert\Delta\text{MW}\rvert$	3.8040	3.9290	-0.1251 ± 3.6787	0.144	ns
		QED	maximize ΔQED	0.1001	0.0945	0.0056 ± 0.0313	1.86×10^{-8}	Original
E	793	QED	minimize QED drop	0.0328	0.0361	-0.0033 ± 0.0242	6.23×10^{-5}	Original
		SA	maximize $-\Delta$SA	0.4421	0.4314	0.0107 ± 0.1523	0.0244	Original
F	793	QED	minimize QED drop	0.0345	0.0329	0.0015 ± 0.0313	<u>0.0823</u>	<u>Ablation</u>
		SA	maximize $-\Delta$SA	0.7233	0.6903	0.0330 ± 0.1598	4.42×10^{-9}	Original

Supplementary Table 3: Summary statistics for the ablation study in Supplementary Figure 4. Original and ablation columns report objective-aligned Δ values averaged over paired input molecules. Paired differences are computed as original minus ablation (mean ± SD). For minimization objectives, negative paired differences favor the original model; for maximization objectives, positive paired differences favor the original model. P-values are obtained from one-sided paired t-tests. By default, the alternative hypothesis tests whether the original model outperforms the ablation. For underlined entries, the direction of the alternative hypothesis is reversed to test whether the ablation outperforms the original; therefore, smaller P-values in these cases indicate stronger evidence in favor of the ablation model. The "Preferred" column reports the model favored by the statistical test, with significance thresholds defined as ns $P > 0.1$, *$P < 0.1$, **$P < 0.05$, ***$P < 0.01$, and ****$P < 0.001$.

(Supplementary Table 3 caption) *"Original and ablation columns report objective-aligned Δ values averaged over paired input molecules. Paired differences are computed as original minus ablation (mean ± SD). P-values are obtained from one-sided paired t-tests."*

<u>Q2</u>. On page 21, the meaning of C_i (with an index) should be clarified.

<u>Reply</u>: We thank the reviewer for pointing out this ambiguity. We clarified the meaning of the indexed condition vector C_i in the Methods section. Specifically, we now explain that C_i

denotes the condition vector assigned to atom i when constructing the input atomic embedding vector. This clarification has been added to the relevant paragraph describing the atom-level embedding formulation.

(Manuscript page 21) *"An initial atom feature vector, â, is concatenated with the condition vector, C, to yield an input atomic embedding vector by $a_i^0 = [\hat{a}_i \| C_i]$. Here, C_i denotes the property-control condition vector assigned to atom a_i, obtained by broadcasting the molecule-level condition vector C to each atom."*

Q3. Typo in Section 5.1 of the Supplementary Information: "proceduer"

Reply: We thank the reviewer for carefully checking the Supplementary Information. We have corrected this typographical error.

(SI page 9) *"We trained a DeepBioisostere model ablated with the subgraph AMPN during the original molecule embedding procedure; in this model the subgraph AMPN is not shared across the molecule and fragment library embeddings, but only used for the fragment library."*

Reviewer #2: Remarks on code availability.

Q1. The code repository has been updated and now includes model parameters and processed datasets.

Reply: We thank the reviewer for confirming the updated code availability. In this revision, we have further prepared an updated Source Data file to support the numerical values underlying the relevant display items, including the newly added statistical summaries. We believe that these additions further improve the transparency and reproducibility of the manuscript.

Reviewer #3

Q1. The authors adequately addressed my previous concerns.

Reply: We sincerely thank the reviewer for their time and positive assessment. We are grateful that the reviewer found our previous revisions sufficient to address their concerns.
